# MicroRNA-378 limits activation of hepatic stellate cells and liver fibrosis by suppressing Gli3 expression

**DOI:** 10.1038/ncomms10993

**Published:** 2016-03-22

**Authors:** Jeongeun Hyun, Sihyung Wang, Jieun Kim, Kummara Madhusudana Rao, Soo Yong Park, Ildoo Chung, Chang-Sik Ha, Sang-Woo Kim, Yang H. Yun, Youngmi Jung

**Affiliations:** 1 Department of Integrated Biological Science, College of Natural Science, Pusan National University, Pusan 46241, Korea; 2 Department of Polymer Science and Engineering, College of Engineering, Pusan National University, Pusan, 46241, Korea; 3 Department of Biological Sciences, College of Natural Science, Pusan National University, 63-2 Pusandaehak-ro, Kumjeong-gu, Pusan 46241, Korea; 4 Department of Biomedical Engineering, College of Engineering, The University of Akron, Akron, Ohio 44685-0302, USA

## Abstract

Hedgehog (Hh) signalling regulates hepatic fibrogenesis. MicroRNAs (miRNAs) mediate various cellular processes; however, their role in liver fibrosis is unclear. Here we investigate regulation of miRNAs in chronically damaged fibrotic liver. MiRNA profiling shows that expression of miR-378 family members (miR-378a-3p, miR-378b and miR-378d) declines in carbon tetrachloride (CCl_4_)-treated compared with corn-oil-treated mice. Overexpression of miR-378a-3p, directly targeting Gli3 in activated hepatic stellate cells (HSCs), reduces expression of Gli3 and profibrotic genes but induces *gfap*, the inactivation marker of HSCs, in CCl_4_-treated liver. Smo blocks transcriptional expression of miR-378a-3p by activating the p65 subunit of nuclear factor-κB (NF-κB). The hepatic level of miR-378a-3p is inversely correlated with the expression of Gli3 in tumour and non-tumour tissues in human hepatocellular carcinoma. Our results demonstrate that miR-378a-3p suppresses activation of HSCs by targeting Gli3 and its expression is regulated by Smo-dependent NF-κB signalling, suggesting miR-378a-3p has therapeutic potential for liver fibrosis.

## Abstract (web summary)

Liver fibrosis is a pathogenic driver of many liver diseases, so understanding its regulation might open the door to new therapies. Here the authors perform a screen for miRNA candidates and identify that miR-378 inhibits liver fibrosis in mice by interfering with Hedgehog signalling in hepatic stellate cells.

## Graphical abstract

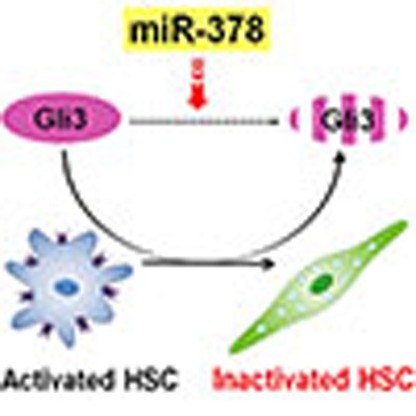

Chronic liver diseases such as cirrhosis and liver cancer are a leading cause of death worldwide^1^. Liver fibrosis, a major characteristic of most chronic liver diseases, accelerates the progression of liver disease to the chronic form by destroying the normal hepatic parenchyma^2^. Therefore, understanding fibrogenesis is important for the development of effective antifibrotic therapies.

MicroRNAs (miRNAs) are endogenous non-coding RNAs of ∼22 nucleotides in length, which are conserved across species^3^. They recognize their target genes through base pairing between a sequence of around six to eight nucleotides in length at their 5′-end (called the seed sequence) and the complementary sequence within the messenger RNA of target genes, suppressing the expression of these target genes at the post-transcriptional level by mRNA decay or by translational inhibition via an miRNA-containing RNA-induced silencing complex^4^. Mammalian miRNAs have been implicated in various biological processes, such as differentiation, proliferation, oxidative stress resistance and tumour suppression^5^,^6^. In addition, miRNAs are known to be dysregulated in several diseases, including cancers. Because of their gene-regulating ability, miRNAs are attractive therapeutic targets. However, most research has focused on the functions of miRNAs in cancer and little is known about the mechanisms of how miRNAs are involved in hepatic pathological processes, including fibrosis.

The Hedgehog (Hh) signalling pathway promotes liver fibrosis^7^,^8^. The Hh ligands, Sonic Hh, Indian Hh and Desert Hh, bind to the Hh receptor Patched (Ptc) and release Smoothened (Smo) into the cytoplasm. Cytoplasmic Smo translocates the Glioblastoma (Gli) family proteins (including Gli1, Gli2 and Gli3) into the nucleus, where they act as transcriptional activators of Hh signalling^8^. In the damaged liver, hepatocytes undergoing apoptosis secrete Hh ligands that initiate proliferation of hepatic progenitor cells and hepatic stellate cells (HSCs)^9^,^10^. These cells in turn produce Hh ligands and further accelerate Hh signalling in an autocrine and paracrine manner. During this process, Hh signalling promotes activation of quiescent HSCs into myofibroblastic HSCs, which contribute to the accumulation of fibrous extracellular matrix (ECM) in the liver^11^,^12^. Hence, Glis are good targets for biomarker and therapeutic approaches aimed at controlling activation of HSCs during liver fibrogenesis.

Here we investigate the involvement of miRNAs in the progression of liver fibrosis, with miRNA array analysis of chronically damaged and fibrotic mouse livers. MiR-378a-3p and its family members, miR-378b and miR-378d, are the most significantly downregulated miRNAs in carbon tetrachloride (CCl_4_)-treated livers compared with corn-oil-treated (control) livers. The miR-378 family is downregulated during HSC activation and suppresses activation of HSCs by targeting *gli3*. In addition, the effect of miR-378a-3p on *gli3* is mediated by transcriptional regulation of the nuclear factor-κB (NF-κB) p65 subunit, interacting with Smo. To explore the clinical implications, we encapsulate miR-378a-3p with nanoparticles (NPs) fabricated with L-tyrosine polyurethane (LTU2a), a biodegradable polymer composed of a modified amino acid, PEG and hexamethylene diisocynate in its backbone^13^,^14^. *In-vivo* application reduces the expression of Gli3 and matrix markers, and leads to the recovery of fibrosis. These results suggest that miR-378 targeting Hh signalling is a novel diagnostic and therapeutic target for understanding and treating the pathogenesis of chronic liver disease.

## Results

### MiR-378 is repressed by CCl_4_-induced chronic liver injury

CCl_4_ is a hepatotoxic chemical that effectively induces liver fibrosis in experimental rodent models^15^. To generate the experimental model of hepatic fibrosis, we exposed mice to CCl_4_ twice a week for 6 (*n*=5) and 10 (*n*=6) weeks by intraperitoneal (i.p.) injection. As a control group for comparison, an equal number of mice were treated with corn oil. Compared with corn-oil treatment, CCl_4_ treatment increased the ratio of liver to body weight in mice (Supplementary Fig. 1a). Haematoxylin and eosin (H&E) staining showed that in liver from CCl_4_-injected mice, there were increases in centrilobular necrosis and the number of apoptotic hepatocytes, and that a large number of immune cells had infiltrated into the hepatic parenchyma (Supplementary Fig. 1b). The RNA level of α-smooth muscle actin (*α-sma*) and collagen type 1-α1 (*col1α1*), and the protein level of Tgf-β and α-SMA were higher in livers of CCl_4_-treated mice than in the liver of control mice (corn-oil-treated mice) (Supplementary Fig. 2a–c). Sirius red stain revealed deposition of excessive collagen fibres in CCl_4_-treated livers and the hydroxyproline content was higher in livers of CCl_4_-treated mice than in the liver of own control mice (Supplementary Fig. 2d,e). In addition, immunostaining for α-SMA, an activated HSC (aHSC) marker^16^, showed that myofibroblastic HSCs had proliferated along the fibrotic tract (Supplementary Fig. 2f). These results confirmed that CCl_4_ induces severe hepatic fibrosis in mice.

**Figure 1:**
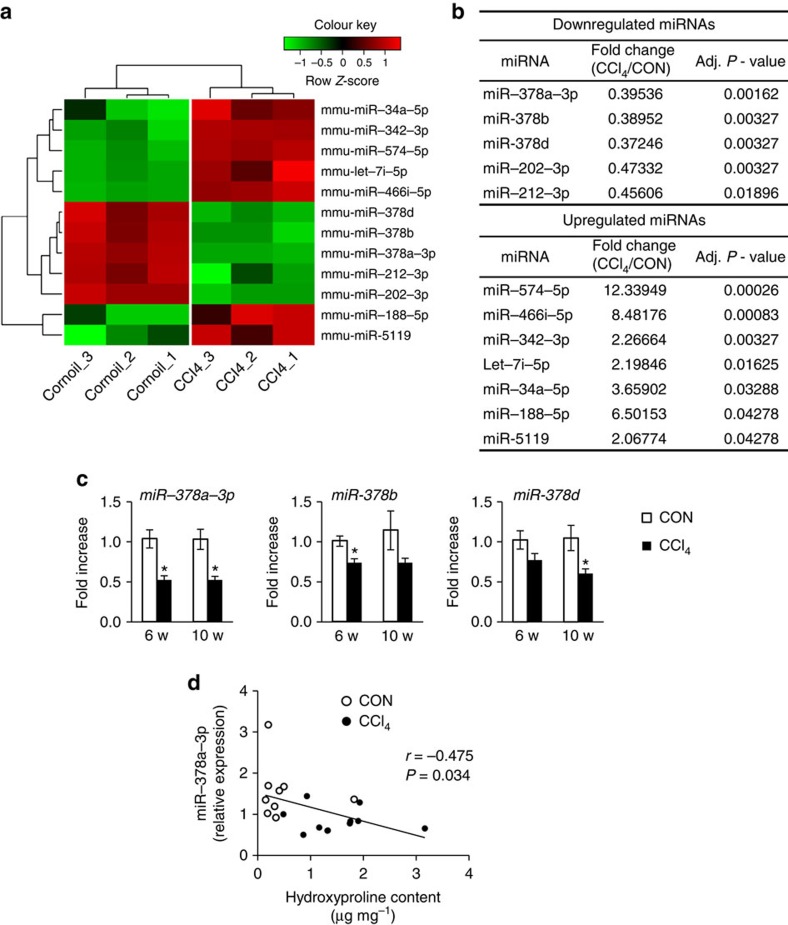
MiR-378 family was downregulated in injured livers of CCl_4_-treated mice. (**a**) Microarray analysis for miRNA expression was performed with total RNA extracted from livers of mice treated with corn-oil (control; CON) or CCl_4_ (*n*=3 per group) for 10 weeks. Heat map shows the two-way hierarchical clustering of differentially expressed miRNAs. Each row and column represents an miRNA and a condition, respectively. The row *Z*-score scaling for the expression level of each miRNA was calculated by subtracting the mean expression of the miRNA from its expression value and then dividing by the s.d. across all the samples. The closer the colour is to bright green, the lower the expression; the closer to bright red, the higher the expression. (**b**) A list of significantly dysregulated miRNAs in CCl_4_-treated compared with corn-oil-treated livers (CON) is shown, with the fold change and *P*-values. (**c**) qRT–PCR was performed to assess expression of the miR-378 family, including miR-378a-3p, miR-378b and miR-378d, in livers from the CON- and CCl_4_-treated mice at 6 and 10 weeks (*n*=4 per group). Mean±s.e.m. results are graphed (unpaired two-sample Student's *t*-test, *P<0.05 versus CON). (**d**) Spearman's rank correlation between miR-378a-3p expression and hydroxyproline contents in liver of all mice (*n*=20, Spearman's rank correlation analysis; *r*, correlation coefficient).

To investigate the differences in miRNA expression profiles between fibrotic and normal livers, we performed miRNA microarrays for total RNA extracts isolated from mouse livers treated with CCl_4_ or corn oil for 10 weeks (*n*=3 per group). MiRNAs were considered to have significant differences in expression level when the expression difference showed more than twofold change between the experimental and control groups at *P*<0.05. We found that 12 miRNAs were differentially expressed in fibrotic liver. Seven miRNAs (mmu-miR-574-5p, mmu-miR-466i-5p, mmu-miR-342-3p, mmu-let7i-5p, mmu-miR-34a-5p, mmu-miR-188-5p and mmu-miR-5119) were upregulated and the other five (mmu-miR-378a-3p, mmu-miR-202-3p, mmu-miR-378b, mmu-miR-378d and mmu-miR-212-3p) were downregulated in the CCl_4_ group compared with the control (Fig. 1a,b). Among these miRNAs that were dysregulated in liver fibrosis, several miR-378 family members, including miR-378a-3p (0.395-fold), miR-378b (0.390-fold) and miR-378d (0.372-fold), had the lowest expression in the livers of CCl_4_-treated mice. This reduced expression of miR-378 family members was validated by real-time quantitative reverse transcriptase–PCR (qRT–PCR) analysis, which showed that miR-378a-3p was significantly decreased, and miR-378b and miR-378d tended to be reduced in fibrotic livers compared with controls (*n*=4 per group) both at 6 and 10 weeks (unpaired two-sample Student's *t*-test, *P*<0.05) (Fig. 1c). In addition, decreased expression of miR-378a-3p in these mice was significantly correlated with the degree of liver fibrosis, as assessed by hydroxyproline assay (Spearman's rank correlation analysis, *r*=−0.475, *P*=0.034, *n*=20) (Fig. 1d).

Microarray and qRT–PCR analyses showed that the expressional level of miR-378a-5p was not significantly changed in fibrotic liver, although it was transcribed together with miR-378 a-3p (Supplementary Fig. 3). MiR-378a-5p was upregulated and influenced SULT2A1 expression in primary sclerosing cholangitis (PSC)^17^. Wunsch *et al*.^17^ demonstrated that PSC is characterized by disease-specific impairment of SULT2A1 expression, which is regulated by miR-378a-5p. These findings suggest that expressional pattern of miR-378a-5p seems to be PSC specific. In line with their findings, miR-378a-3p, not the 5p, shows the significant changes in CCl_4_-treated mice and the association with HSC activation. These findings suggest the possibility that miR-378a-3p and miR-378a-5p have disease-specific effects in liver. Hence, further study is needed to investigate the potential role of miR-378 in PSC.

### Expression of miR-378 is reduced during HSC activation

**Figure 2:**
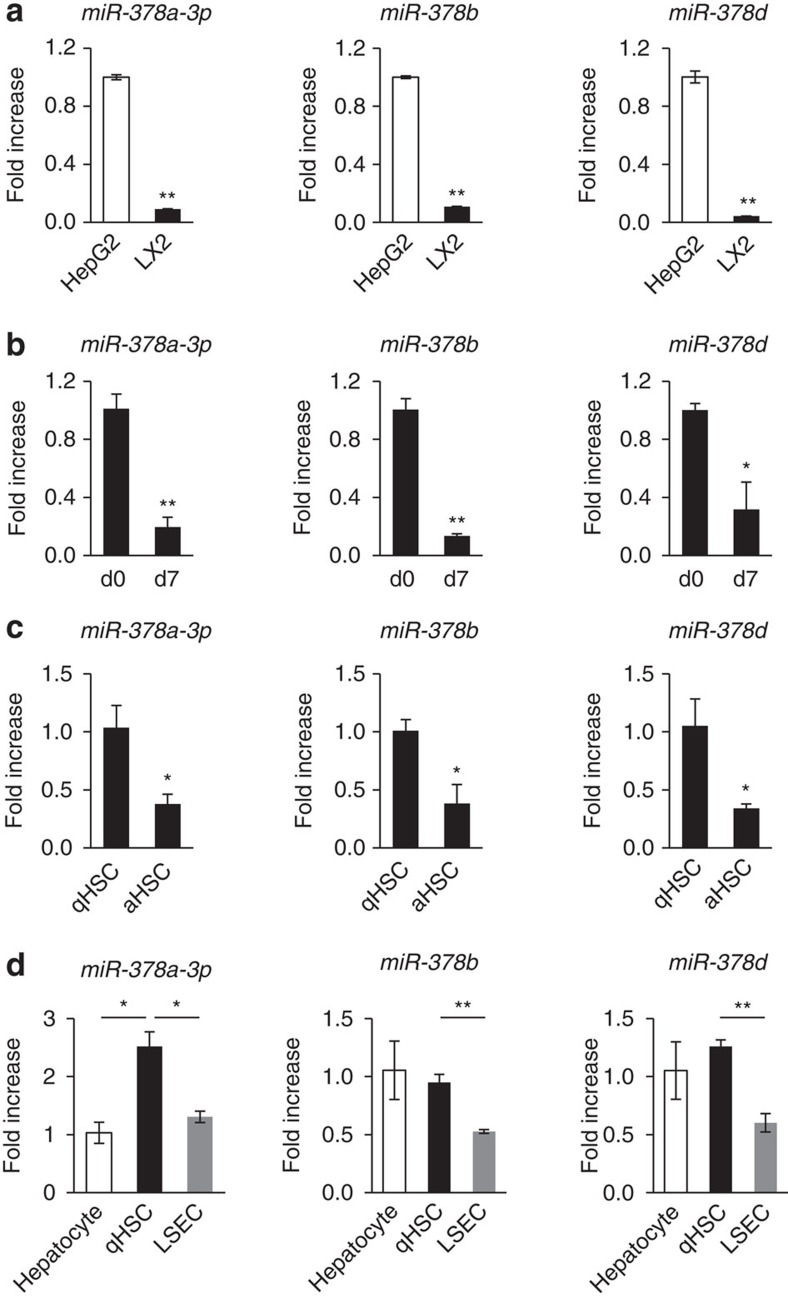
Decreased expression of the miR-378 family in aHSCs. (**a**) Expression of miR-378 family members, including miR-378a-3p, miR-378b and miR-378d, in HepG2 (human liver epithelial cell line) and LX2 (human aHSC line) cells was examined by qRT–PCR. (**b**) qRT–PCR of the miR-378 family in primary qHSCs isolated from normal C57BL/6 mice at quiescent stage (d0; immediately after isolation) and in primary aHSCs (d7; cultured for 7 days). (**c**) qRT–PCR of the miR-378 family in primary HSCs isolated from corn-oil- (qHSCs) or CCl_4_-(aHSCs)-treated mice. (**d**) Expression of the miR-378 family in primary hepatocytes, primary qHSC and LSECs isolated from normal C57BL/6 mice, as assessed by qRT–PCR. All results of relative expression values are shown as mean±s.e.m. of triplicate experiments (unpaired two-sample Student's *t*-test, *P<0.05 and **P<0.005).

As the aHSC is a major ECM-producing cell contributing to liver fibrosis^18^,^19^, we examined the expression of miR-378 family members in LX2, a human aHSC line. Expression of all of three miR-378 family members was reduced to a greater extent in LX2 than in HepG2, a human hepatocyte cell line (Fig. 2a). As primary HSCs are known to be activated during culture^20^,^21^, we isolated primary HSCs from mice and assessed whether the expression level of these miRNAs was changed during HSC culture. We found that miR-378a-3p, miR-378b and miR-378d were significantly downregulated at day 7 compared with day 0 of culture (Fig. 2b). To confirm these findings *in vivo*, we isolated primary HSCs from corn-oil- or CCl_4_-treated mice (Supplementary Figs 1 and 2). The expression of miR-378a-3p, miR-378b and miR-378d was also lower in primary aHSCs isolated from CCl_4_-treated mice than inactivated cells from corn-oil-treated mice (Fig. 2c). Those results indicate that these miRNAs were downregulated during HSC activation. In addition, we examined the expression of miR-378 family members in different types of liver cells, including hepatocytes, quiescent (day 0) HSCs (qHSCs) and liver sinusoidal endothelial cells (LSECs), primarily isolated from livers of normal mice. Quiescent HSCs contained a higher level of miR-378a-3p and similar levels of miR-378b and miR-378d compared with hepatocytes. Expression of all three miR-378 family member was greater in qHSCs than in primary LSECs (Fig. 2d). These results suggested that the miR-378 family is involved in HSC activation.

### MiR-378a-3p directly binds to Gli3 mRNA

**Figure 3:**
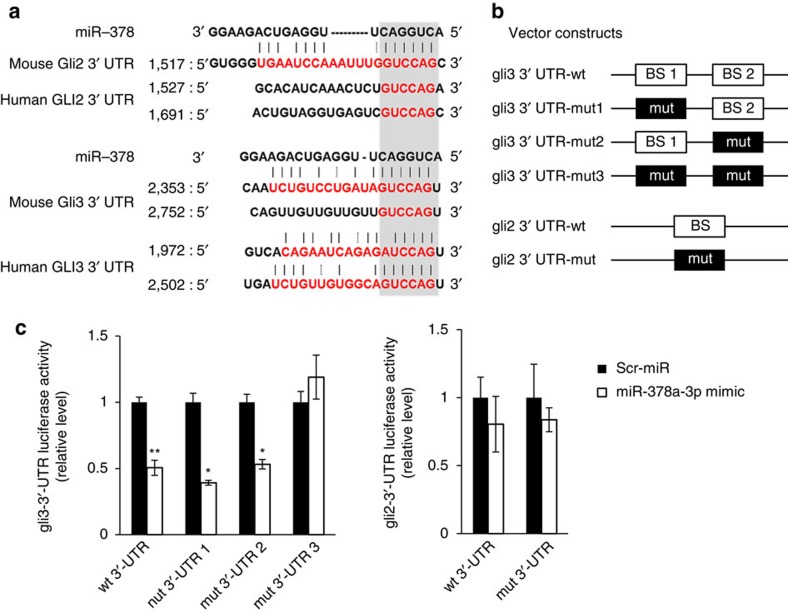
MiR-378 binds directly to Gli3. (**a**) Using an miRNA database (www.miRNA.org), putative binding sites (red font) of miR-378a-3p were predicted in the 3′-UTR of *gli2* and *gli3* mRNA in mouse and human. The dashed line represents complementary base pairs between miR-378a-3p and *gli2* or *gli3* mRNA, whereas the grey shading indicates the seed sequence of miR-378a-3p. (**b**) psiCHECK-2 vectors containing either the wild-type (wt) or mutant (mut) binding site of miR-378a-3p in *gli2* and *gli3* mRNAs were constructed to conduct luciferase reporter assays. The mutated nucleotides are shown in Supplementary Fig. 6. (**c**) Dual luciferase reporter assay was performed to verify binding between miR-378a-3p and *gli2* or *gli3* mRNA. N2a, a mouse neuroblastoma cell line, was co-transfected with a psiCHECK-2 vector containing either the wt or mut target sites plus either the miR-378a-3p mimic or the scrambled (Scr)-miR (control) oligonucleotide. Results of relative luciferase activity are shown as mean±s.e.m. obtained from triplicate experiments (unpaired two-sample Student's *t*-test, *P<0.05, **P<0.005 versus Scr-miR).

To identify the relevant target genes of the miR-378 family, we conducted bioinformatic analysis using http://www.microrna.org/, a comprehensive resource of miRNA target predictions^22^. We found that *gli2* and *gli3*, downstream transcriptional factors of Hh signalling, were potential targets of miR-378a-3p, both in human and mouse, two species that share common miR-378a-3p seed sequences (Fig. 3a). Hence, we investigated whether miR-378a-3p interacted with the binding sites at the 3′-untranslated region (3′-UTR) of *gli2* and *gli3* mRNAs (Fig. 3b) using luciferase assay in neuroblasoma cell line (N2a). To fit the species of the experimental animals, we employed this cell line originated from mouse. Luciferase reporter assay revealed that miR-378a-3p, not miR-378b or miR-378d, directly bound to *gli3* but not *gli2* mRNA, and that both of the two binding sites of the *gli3* mRNA were active (Fig. 3c and Supplementary Fig. 4). Expression of *Gli3* and *Gli2* at both the RNA and protein levels was elevated, whereas expression of *gli1* was reduced in livers of CCl_4_-treated mice (Supplementary Fig. 5a–c). Pritchett *et al*.^23^ also demonstrated that *gli1* is sparse in HSCs and does not increase on activation of HSCs. Interestingly, the amount of miR-378a-3p expression was inversely correlated with the hepatic level of Gli3 expression in those mice (Spearman's rank correlation analysis, *r*=−0.733, *P*=0.007, *n*=12) (Supplementary Fig. 5d). In addition, LX2 cells showed a larger increase in *gli3* and *gli2*, compared with HepG2 cells (Supplementary Fig. 5e). These results suggest that upregulation of *Gli3* expression is related to significant reduction of miR-378a-3p targeting *gli3* in chronically damaged liver.

### MiR-378a-3p suppresses activation of HSCs

**Figure 4:**
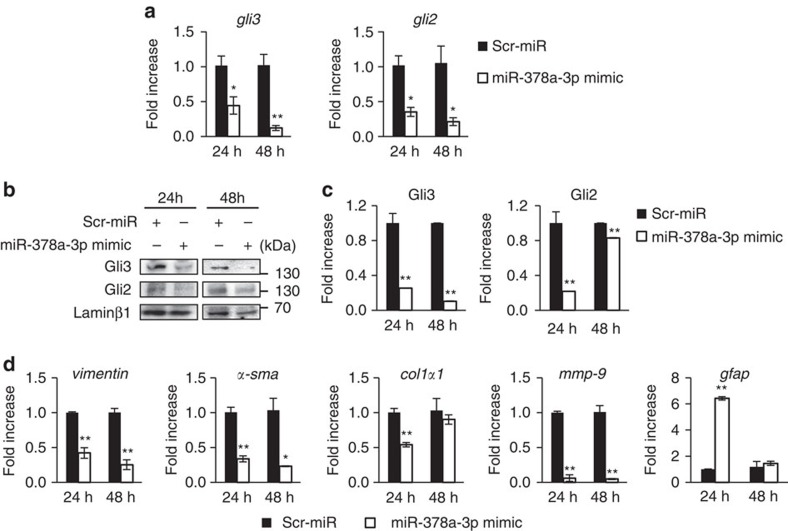
MiR-378a-3p induces inactivation of primary HSCs by reducing Gli3 and Gli2 expression. (**a**) Primary aHSCs were transfected with either an miR-378a-3p mimic (white bar) or scrambled (Scr)-miR (control) (black bar) oligonucleotide for 24 and 48 h, and expression of *gli3* and *gli2* was assessed by qRT–PCR. (**b**) Western blot analysis and (**c**) cumulative densitometric analyses for nuclear Gli3 (145 kDa) and Gli2 (133 kDa), with Laminβ1 (68 kDa) as an internal control for nuclear fraction. Data shown represent one of three experiments with similar results. (**d**) qRT–PCR analysis for genes related to activation of HSC, including *vimentin*, *α-sma*, *col1α1* and *mmp9*, and the inactivation marker of HSC, *gfap*, in primary HSCs transfected with miR-378a-3p mimic (white bar) or scrambled (Scr)-miR (control) (black bar) oligonucleotide for 24 and 48 h. All results of relative expression values are shown as mean±s.e.m. obtained from triplicate experiments (unpaired two-sample Student's *t*-test, *P<0.05 and **P<0.005 versus Scr-miR).

We investigated whether ectopic expression of miR-378a-3p in the aHSCs influenced HSC activation. Therefore, we isolated primary aHSCs from CCl_4_-treated mice and transfected these cells with miR-378a-3p mimic or scrambled-miR (negative control), and then analysed the expression levels of genes related to HSC activation at 24 and 48 h after transfection. As expected, *Gli3* at both the mRNA and protein levels was significantly downregulated in miR-378a-3p mimic-transfected cells, as analysed by qRT–PCR and western blotting (unpaired two-sample Student's *t*-test, *P*<0.05) (Fig. 4a–c). Although miR-378a-3p was shown to be unbound to the *gli2* mRNA (Fig. 3c), the miR-378a-3p mimic also reduced the expression of Gli2 at 24 h, implying that miR-378a-3p was indirectly regulating Gli2 expression through the interaction between Gli3 and Gli2 (Fig. 4a–c). In addition, in primary aHSCs transfected with a miR-378a-3p mimic, expression of the profibrotic genes encoding for Vimentin, α-SMA, Col1α1 and Mmp-9 decreased, whereas expression of glial fibrillary acidic protein (*gfap*), an inactivation marker of HSCs^16^,^24^, increased (Fig. 4d). In LX2 cells transfected with miR-378a-3p mimic, similar results were observed; the level of *Gli3* and *Gli2* was reduced and the expression of profibrotic markers, *vimentin*, plasminogen activator inhibitor-I (*pai-I*) and connective tissue growth factor (*ctgf*) was also downregulated^25^, but *gfap* was upregulated (Supplementary Fig. 7). Therefore, these results demonstrate that miR-378a-3p suppresses the activation of HSCs by directly targeting *gli3*.

### Smo regulates expression of miR-378

**Figure 5:**
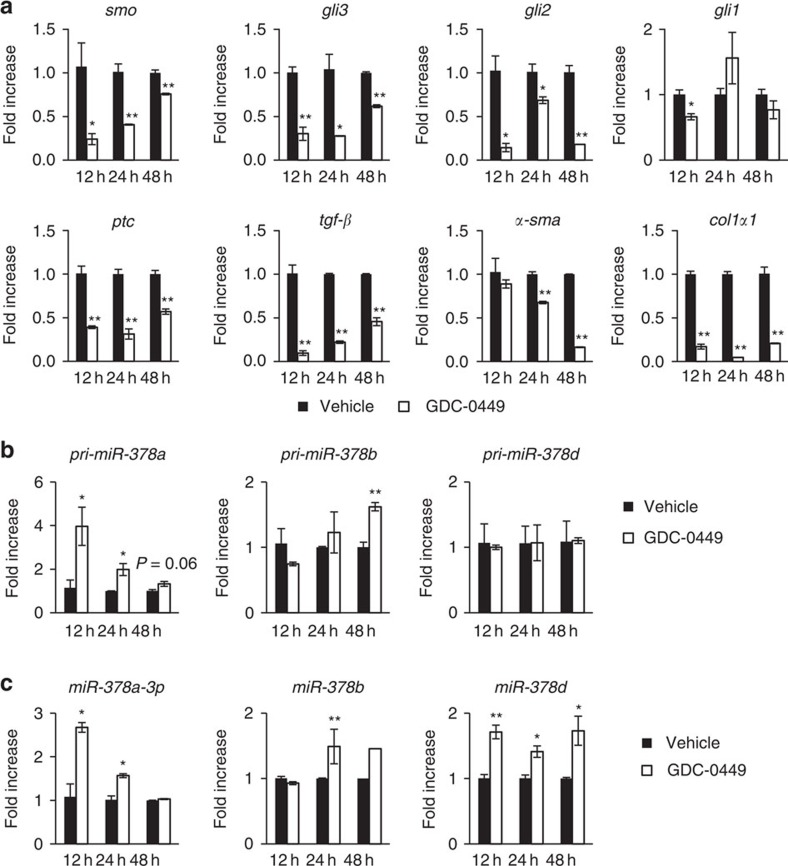
Smo influences expression of miR-378. (**a**) qRT–PCR of the expression of Hh signals, including *smo*, *gli2*, *gli3*, *gli1* and *ptc*, and profibrotic genes, including *tgf-β*, *α-sma* and *col1α*, in LX2 cells treated with (white bar) or without (black bar) 1 μM of GDC-0449, a Smoothened (Smo) antagonist, for 12, 24 and 48 h. (**b**) qRT–PCR analysis of miR-378a precursor (pri-miR-378a), miR-378b precursor (pri-miR-378b) and miR-378d precursor (pri-miR-378d) in LX2 cells treated with (white bar) or without (black bar) GDC-0449 for 12, 24 and 48 h. (**c**) qRT–PCR analysis of mature family members, including miR-378a-3p, miR-378b and miR-378d, in LX2 cells treated with (white bar) or without (black bar) GDC-0449 for 12, 24 and 48 h. All results of relative expression values are shown as mean±s.e.m. obtained from triplicate experiments (unpaired two-sample Student's *t*-test, *P<0.05 and **P<0.005 versus vehicle).

As miR-378a-3p targets Gli3 and the Glis are activated by stimulated Hh receptors, it is possible that Smo influences expression of miR-378a-3p. To investigate this possibility, we treated fully activated LX2 cells with GDC-0449, an Smo antagonist, for 12, 24 and 48 h. As expected, expression of the Hh receptors, *ptc* and *smo*, and the Hh target genes, *gli1*, *gli2* and *gli3*, decreased in LX2 cells treated with GDC-0449. The fibrotic markers, *tgf-β*, *α-sma* and *col1α1*, were also reduced in GDC-0449-treated LX2 cells (Fig. 5a). Interestingly, inhibition of Smo induced the upregulation of pri-miR-378a and miR-378a-3p at 12 and 24 h post GDC-0449 treatment (Fig. 5b,c). As this inhibitory effect of GDC-0449 was alleviated, the level of *smo* gradually increased. In parallel with Smo elevation, the expression of pri-miR-378a and miR-378a-3p in GDC-0449-treated LX2 cells gradually decreased and was similar with it in vehicle-treated LX2 cells. The expressional changes of precursor and mature form of miR-378b and miR-378d were subtle or rare in Smo inhibitor-treated cells, indicating that Smo poorly influenced their expression. As these miRNA are located on different chromosomes, they seem to show the different response to Smo inhibitor.

**Figure 6:**
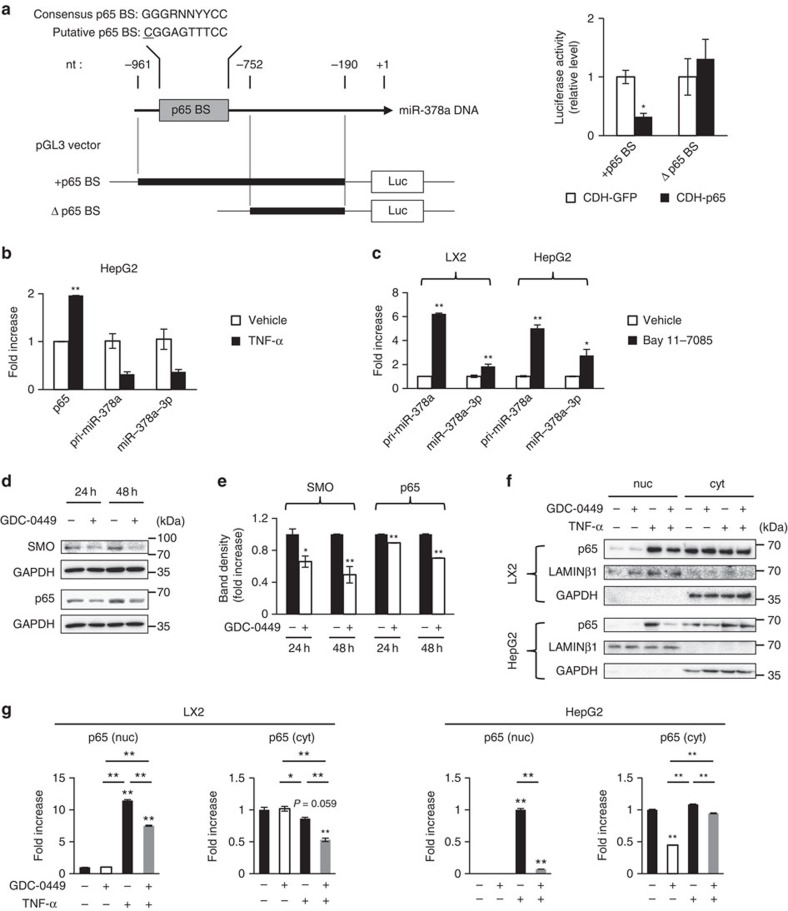
Expression of miR-378a is regulated by Smo-dependent activation of p65. (**a**) p65 binding site (p65BS, grey box) on miR-378a DNA (arrow line) and inserted regions (bold line) into pGL3-basic vector are depicted. The consensus and putative binding sequences predicted by TRANSFAC are shown; underlining indicates a mismatched nucleotide (nt). pGL3-basic vectors with (+p65BS) or without (Δp65BS) a p65-binding site on the promoter region of pri-miR-378a were constructed. HepG2 cells were co-transfected with +p65BS and either CDH-p65 (black) or CDH-GFP (control) (white), and a separate group of HepG2 cells were co-transfected with Δp65BS and either CDH-p65 (black) or CDH-GFP (white). Results of relative luciferase activity are shown as mean±s.e.m. obtained from triplicate experiments (unpaired two-sample Student's *t*-test, *P<0.05 versus CDH-GFP). Luc, luciferase. (**b**) Expression level of *p65* mRNA, pri-miR-378a and miR-378a-3p in HepG2 treated with 100 ng ml^−1^ of TNF-α (black) or vehicle (white) was analysed by qRT–PCR. Relative expression is shown as mean±s.e.m. obtained from triplicate experiments, compared with HepG2 treated with vehicle (unpaired two-sample Student's *t*-test, *P<0.05 and **P<0.005 versus vehicle). (**c**) qRT–PCR analysis for pri-miR-378a and miR-378a-3p in LX2 or HepG2 cells treated with 2 μM of Bay 11-7085 (black) or vehicle (white). Results are shown as mean±s.e.m. obtained from triplicate experiments (unpaired two-sample Student's *t*-test, **P<0.005 versus vehicle). (**d**) Western blot analysis for SMO (86 kDa), p65 (65 kDa) and GAPDH (36 kDa) in LX2 cells treated with GDC-0449 (1 μM) or vehicle for 24 and 48 h. A representative image from triplicate experiments is shown. (**e**) Cumulative densitometric analyses of SMO and p65 western blotting results are displayed as the mean±s.e.m. (unpaired two-sample Student's *t*-test, *P<0.05 and **P<0.005 versus vehicle). (**f**) Western blot assay for p65, Laminβ1 (68 kDa) and GAPDH in vehicle- or TNF- and/or GDC-0449-treated LX2 and HepG2 cells. Laminβ1 and GAPDH were used as internal controls for the nuclear (nuc) and cytosolic (cyt) fractions, respectively. Data shown represent one of three experiments with similar results. (**g**) Cumulative densitometric analyses of SMO and p65 western blotting results are displayed as the mean±s.e.m. (one-way analysis of variance (ANOVA) with Tukey corrections, *P<0.05 and **P<0.005 versus vehicle-treated control).

This elevated expression of pri-miR-378a by Smo suppression suggested that Smo might influence the level of pri-miR-378a transcription. Hence, using a prediction system, the TRANSFAC programme, we examined whether Smo might bind the gene of *miR-378a*. The data indicated that an upstream sequence of *miR-378a*, from nucleotides −820 to around −811, contained a putative binding site for NF-κB (p65), rather than Smo, at the 5′-region (Fig. 6a). Luciferase reporter assay was conducted to assess the functional binding of p65 in this region of the *miR-378a* gene. We constructed a pGL3 vector with (+p65BS) or without (Δp65BS) a p65-binding site and co-transfected HepG2 cells with this vector and either a CDH-p65 vector or a CDH-GFP (control) vector. Luciferase activity was lower in +p65BS-transfected HepG2 cells with CDH-p65 than with CDH-GFP vector. However, there was no significant difference in luciferase activity between Δp65BS/CDH-GFP-transfected and Δp65BS/CDH-p65-transfected HepG2 cells. Thus, the results of the luciferase reporter assay confirmed that p65 directly binds to the *miR-378a* gene (Fig. 6a).

To investigate whether activation of NF-κB signalling influences expression of miR-378a-3p, we treated cells with either an NF-κB signalling activator or repressor. Treatment with tumour necrosis factor-α (TNF-α), an activator of NF-κB signalling, induced upregulation of *p65* but downregulation of pri-miR-378a and miR-378a-3p in HepG2 cells (Fig. 6b). In TNF-α-treated LX2 cells, expression of miR-378a-3p was not significantly changed (data now shown). As miR-378a-3p is greatly downregulated in LX2 cells, the inhibitory effect of p65 in miR-378a-3p expression seems to be poor. However, the expression level of pri-miR-378a and miR-378a-3p was higher in both LX2 and HepG2 cells treated with the NF-κB inhibitor, Bay 11-7085, than in vehicle-treated cells (Fig. 6c). When miRNA expression is simultaneously induced, the level of pri-miRNAs reaches its peak earlier than mature miRNAs do^26^. Similar to these findings, pri-miR-378a in NF-κB inhibitor-treated LX2 or HepG2 cells was more upregulated compared with miR-378a-3p at 24 h. These data suggest that p65 functions as a repressor of transcriptional regulation of miR-378a-3p.

To examine how Smo is associated with p65 signalling in the transcriptional regulation of miR-378a-3p, we assessed whether p65 is activated by Smo. The level of p65 protein declined in LX2 cells at 24 and 48 h post GDC-0449 treatment (Fig. 6d,e). TNF-α treatment increased the level of the nuclear fraction of p65 in LX2 cells, whereas TNF-α added to the Smo inhibitor-treated LX2 cells led to a reduction in p65 expression (Fig. 6f,g). The level of the cytosolic p65 was slightly reduced in Smo inhibitor and TNF-α-treated LX2 cells, indicating that the amount of p65 protein was generally regulated in these cells. The similar results were also observed in HepG2 cells (Fig. 6f,g). Therefore, these results indicate that Smo promotes nuclear localization of p65, which then binds to the *miR-378a* gene and inhibits transcription of *miR-378a* to pri-miR-378a, suggesting that reduction of miR-378a-3p expression by Smo-activated p65 contributes to increased expression of Gli3 in aHSCs. In previous research, we showed that Hh signalling was associated with radiation-induced hepatic fibrosis, which was attenuated in GDC-0449-treated liver with irradiation^27^. Hence, we checked the expression of miR-378a-3p in the liver of Smo-suppressed mice with radiation. Expression of *p65* decreased in the radiation-treated liver with Smo inhibition, but its expression increased in the radiation-treated liver with fibrosis. Both of pri-miR-378a and miR-378a-3p were downregulated in radiation-treated liver, whereas they were upregulated in irradiated liver treated with Smo inhibitor. Compared with hepatic level of *gli3* in irradiated mice, livers of irradiated mice treated with Smo inhibitor contained lower level of *gli3* (Supplementary Fig. 8). These results supported the regulatory effect of Smo in the expression of miR-378a-3p through p65 activation.

### NP-encapsulated miR-378a-3p reduces hepatic damage

To deliver the miR-378a-3p into experimental animal model, LTU2a NPs encapsulated with miR-378a-3p mimic (NP/M) or scrambled miRNAs (NP/NC) as a negative control were prepared. Transmission electron microscopy of NPs shows relatively spherical morphology and their average sizes are 340 and 516 nm for NP/M and NP/NC, respectively (Supplementary Fig. 9a,b). Zeta potential analysis showed that the overall charge of them was −9.3 and −3.9 mV at peak for NP/M and NP/NC, respectively (Supplementary Fig. 9c). *In vitro* release kinetics show burst release of miRNA for the first 2 days for both formulations (Supplementary Fig. 9d). Afterwards, the NP/M formulation shows a biphasic release profile, whereas the NP/NC does a controlled release phase. The release of miRNAs reached steady state within 5 weeks. To investigate the uptake of the NPs, LX2 cells were cultured in medium and exposed NPs encapsulated with fluorescein isothiocyanate (FITC)-conjugated BSA for overnight. The co-localization of the NPs within the cell by fluorescence and confocal microscopy (Supplementary Fig. 10) demonstrated successful uptake.

To investigate the function of miR-378a-3p on liver fibrosis *in vivo*, we first examined the effect of NPs in the healthy livers. Mice were given once i.p. injection of 6 nmol per mice of NP/NC (*n*=4) or PBS vehicle (*n*=4) for 3 weeks and then killed. In the examination of liver of mice with or without NPs/NC, liver morphology and expression of genes, *miR-378a-3p*, *gli3* and *col1α1*, were similar between two groups (Supplementary Fig. 11a,b). The expression of α-SMA was hardly detected, but the expression of GFAP was greater in both vehicle and NP/NC groups compared with the CCl_4_-treated group (Supplementary Fig. 11c,d). Based on this data, livers of mice with NPs/NC were used for control group in further analysis. NPs/M or NPs/NC were injected via i.p. route of administration into CCl_4_ or corn-oil-treated mice. Mice were killed at 1, 2 and 3 weeks after NPs treatment (*n*=4 per group per week) (Supplementary Fig. 12a). H&E staining showed the severe hepatic injuries in CCl_4_-treated mice with (CCl_4_+NPs/NC group) or without NPs/NC (CCl_4_ group) at 3 weeks post NP injection. Interestingly, those abnormal morphological changes were remarkably ameliorated in the CCl_4_+NPs/M group, compared with the CCl_4_ and CCl_4_+NPs/NC groups (Supplementary Fig. 12b). The ratio of LW/BW of the CCl_4_ and the CCl_4_+NPs/NC group increased at 3 weeks, compared with control and the CCl_4_+NPs/M group (CCl_4_: 1.31±0.028, CCl_4_+NPs/NC: 1.31±0.019, CCl_4_+NPs/M: 1.17±0.044 versus CON, Kruskal–Wallis test and unpaired two-sample Student's *t*-test, *P<0.05and **P<0.005) (Supplementary Fig. 12c). In addition, CCl_4_-treated mice with or without NPs/NC had elevated serum alanine transaminase (ALT) and aspartate transaminase (AST), whereas CCl_4_-treated mice with NPs/M had alleviated ALT and AST at 3 weeks (ALT: CCl_4_: 2.28±0.255, CCl_4_+NPs/NC: 2.98±0.449, CCl_4_+NPs/M: 1.23±0.176 versus CON/AST: CCl_4_: 1.45±0.083, CCl_4_+NPs/NC: 1.47±0.118, CCl_4_+NPs/M: 1.04±0.159 versus CON, Kruskal–Wallis test and unpaired two-sample Student's *t*-test, *P<0.05 and **P<0.005) (Supplementary Fig. 12d). These results suggested that miR-378a-3p mimic ameliorated chronic liver injury induced by CCl_4_ in mice.

### MiR-378a-3p reduces expression of Gli3 and fibrosis in the liver

**Figure 7:**
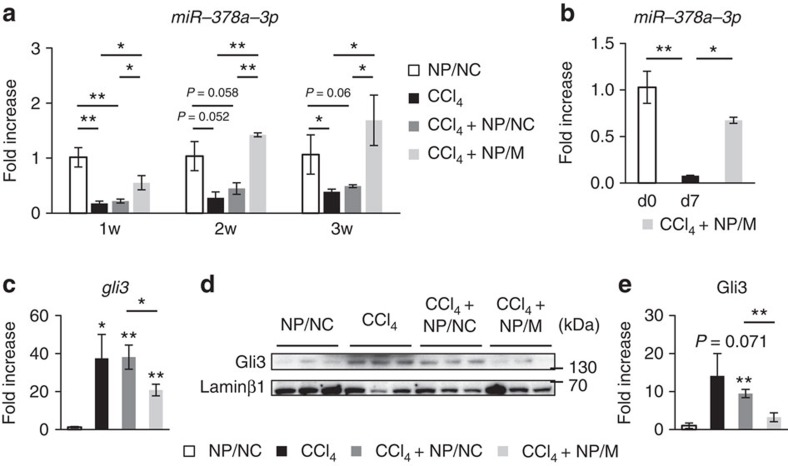
NPs having miR-378a-3p increase the level of miR-378a-3p but decrease Gli3 expression in CCl_4_-treated mice. (**a**) qRT–PCR for *miR-378a-3p* in livers from NP/NC, CCl_4_, CCl_4_-treated with NP/NC (CCl_4_+NP/NC) or NP/M (CCl_4_+NP/M) mice (*n*=4 per group). Mean±s.e.m. results are graphed (Kruskal–Wallis test and unpaired two-sample Student's *t*-test, *P<0.05 and **P<0.005 versus NP/NC). (**b**) qRT–PCR of *miR-378a-3p* in primary qHSCs isolated from normal mice at quiescent stage (d0; immediately after isolation), primary aHSCs (d7; cultured for 7 days) and primary HSCs isolated from CCl_4_+NP/M mice at 3 weeks after NPs treatment. Results of relative expression values are shown as mean±s.e.m. of triplicate experiments (one-way analysis of variance (ANOVA) with Tukey corrections, *P<0.05 and **P<0.005 versus d0). (**c**) qRT–PCR analysis for *gli3* in livers from all mice at 3 weeks after NPs treatment (*n*=4 per group). Mean±s.e.m. results are graphed (Kruskal–Wallis test and unpaired two-sample Student's *t*-test, *P<0.05 and **P<0.005 versus NP/NC). (**d**) Western blot analysis for Gli3 (145 kDa) and Laminβ1 (68 kDa, internal control for nuclear fraction) in livers of three representative mice from each group. Data shown represent one of three experiments with similar results. (**e**) Cumulative densitometric analyses of Gli3 western blotting results are displayed as the mean±s.e.m. (Kruskal–Wallis test and unpaired two-sample Student's *t*-test, **P<0.05).

**Figure 8:**
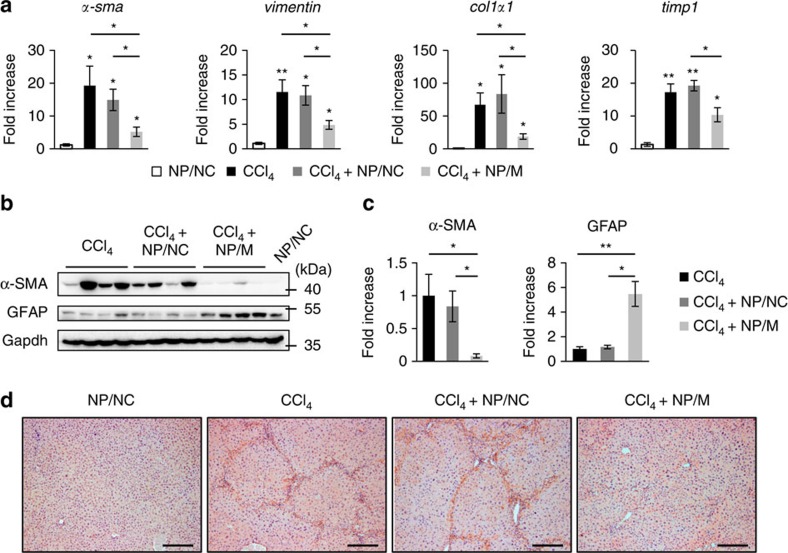
MiR-378a-3p promotes inactivation of HSC in CCl_4_-treated mice. (**a**) qRT–PCR analysis for *α-sma*, *vimentin*, *col1α1* and *timp1* in NP/NC, CCl_4_, CCl_4_+NPs/NC and CCl_4_+NPs/M group (*n*=4 per group). All results of relative expression values are shown as mean±s.e.m. (Kruskal–Wallis test and unpaired two-sample Student's *t*-test, *P<0.05 and **P<0.005 versus NP/NC). (**b**) Western blot analysis for α-SMA (42 kDa), GFAP (50 kDa) and GAPDH (36 kDa) in livers of representative mice from each group. Data shown represent one of three experiments with similar results. (**c**) Cumulative densitometric analyses of α-SMA and GFAP western blotting results are displayed as the mean±s.e.m. (Kruskal–Wallis test and unpaired two-sample Student's *t*-test, *P<0.05 and **P<0.005). (**d**) Immunohistochemistry for α-SMA in liver sections from representative NP/NC, CCl_4_, CCl_4_+NPs/NC and CCl_4_+NPs/M group (scale bar, 100 μm).

To assess whether NPs/M were delivered into livers, the level of hepatic miR-378a-3p was examined by qRT–PCR analysis. Compared with CCl_4_-treated mice with or without NPs/NC, expression of miR-378a-3p was significantly upregulated in CCl_4_-treated mice with NPs/M-injection and gradually increased after NPs/M treatment, suggesting that NPs/M migrated into and stably released RNA oligonucleotides in the liver during this period (Kruskal–Wallis test and unpaired two-sample Student's *t*-test, *P*<0.05) (Fig. 7a). In addition, expression of miR-378a-3p was higher in primary HSCs isolated from livers of mice treated with CCl_4_+NPs/M than in primary aHSCs (day 7) and almost similar to in primary qHSCs (day 0) (Fig. 7b). These findings indicated that NPs/M was successfully taken by HSCs in CCl_4_-injured livers. As most upregulation of miR-378a-3p was observed in CCl_4_-treated liver at 3 weeks post NPs/M treatment, we assessed the expression of *Gli3* and genes related with HSC activation at this time point. The level of *Gli3* in both RNA and nuclear-fractionized protein was downregulated in the CCl_4_+NPs/M group, compared with the CCl_4_ or the CCl_4_+NPs/NC group (Fig. 7c–e). RNA expression of *α-sma* and *vimentin*, *col1α1* and *timp1* was reduced in livers of the CCl_4_+NPs/M group (Fig. 8a). In addition, protein level of α-SMA was lower but level of GFAP, inactivation marker of HSC, was higher in the CCl_4_+NPs/M group than in the CCl_4_ or the CCl_4_+NPs/NC group (Fig. 8b,c). Furthermore, immunostaining for α-SMA confirmed that accumulation of α-SMA-positive HSCs was ameliorated remarkably in the liver of NPs/M-treated mice with CCl_4_ treatment (Fig. 8d). These results demonstrated that miR-378a-3p delivered by NPs led to downregulation of Gli3, which promoted inactivation of HSCs in the livers of CCl_4_-treated mice.

### Level of miR-378 declines in MCDE-fed mice and human HCC

**Figure 9:**
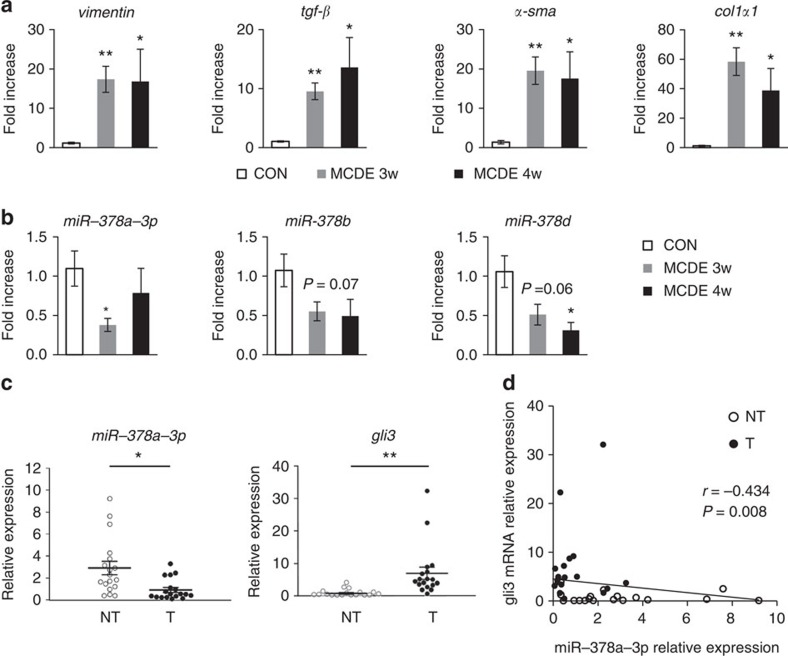
Expression of miR-378 declines in injured liver of MCDE-fed mice and of patients with HCC. (**a**) qRT–PCR of pro-fibrotic markers, *vimentin*, *tgf-β*, *α-sma* and *col1α1*, in mice fed with normal chow (CON) or a methionine/choline-deficient diet supplemented with 0.1% ethionine (MCDE) for 3 and 4 weeks (*n*=4 per group). Mean±s.e.m. results are graphed (unpaired two-sample Student's *t*-test, * *P*<0.05 and ** *P*<0.005 versus CON). (**b**) Expression of miR-378 family members, including miR-378a-3p, miR-378b and miR-378d, in MCDE-treated mice was examined (*n*=4 per group). Mean±s.e.m. results are graphed (unpaired two-sample Student's *t*-test, *P<0.05 versus CON). (**c**) qRT–PCR of miR-378a-3p and *gli3* in paired non-tumour (NT) and tumour (T) regions of liver tissue from patients with human hepatocellular carcinoma (HCC) (*n*=18 patients). Individual values represented by the relative expression are shown by dots and their mean±s.e.m. results presented for each group (paired two-sample Student's *t*-test, *P<0.05 and **P<0.005 versus NT). (**d**) Spearman's rank correlation between miR-378a-3p expression and *gli3* expression in NT and T of HCC (Spearman's rank correlation analysis; *r*, correlation coefficient).

To assess whether this reduction in levels of the miR-378 family also occurs in other types of liver injury models, we examined the expression of miR-378 family members in livers from mice fed with a methionine/choline-deficient diet supplemented with 0.1% ethionine (MCDE), which causes the non-alcoholic steatohepatitis (NASH) that accompanies hepatic fibrosis (*n*=4 per group)^9^. Compared with livers of chow-fed mice, livers of MCDE-fed mice contained significantly increased expression of fibrotic markers including *vimentin*, *tgf-β*, *α-sma* and *col1α1* (Fig. 9a). Expression of miR-378a-3p and miR-378d was significantly lower in the MCDE-fed than the normal chow-fed mice at weeks 3 and 4 (unpaired two-sample Student's *t*-test, *P*<0.05). In addition, miR-378b tended to be downregulated in livers from MCDE-fed mice during liver injury (Fig. 9b).

To investigate the potential role of miR-378a-3p in its clinical implications, we analysed the expression of miR-378a-3p and its target, *gli3*, in non-tumour and tumour regions of liver tissues obtained from 18 patients with hepatocellular carcinoma (HCC) and advanced fibrosis (stage F3 or F4). The level of miR-378a-3p was lower in tumour tissues (0.82±0.185) than in non-tumour tissues (2.90±0.614), whereas *gli3* mRNA level was higher in tumour tissues (6.98±1.991) than in non-tumour tissues (0.76±0.222) (Fig. 9c). As expression of miR-378a-3p was decreased in the aHSCs and fibrotic liver tissue containing higher levels of *gli3*, we performed Spearman's rank correlation analysis to assess the degree of correlation between the levels of miR-378a-3p and *gli3* in non-tumour and tumour liver tissue from individual HCC samples. The amount of *gli3* mRNA was inversely correlated with the hepatic level of miR-378a-3p in those samples (*r*=−0.434, *P*=0.008, *n*=36) (Fig. 9d). These findings support the clinical implications of miR-378a-3p regulating Hh signalling in treating HCC.

## Discussion

MiR-378a is located in the intron of the peroxisome proliferator-activated receptor-γ coactivator 1β of human chromosome 5 and mouse chromosome 18. It is co-expressed with its host gene *PGC-1β* and constitutive activation of miR-378a in hepatocytes has been shown to prevent progression to hepatic fibrosis^28^. It was also reported that the miR-378a-3p was abundant in cardiomyocytes, but was downregulated and induced the upregulation of TGF-β1 during cardiac fibrosis^29^. In line with the findings of that study, our results demonstrated that expression of miR-378a-3p declined during liver fibrosis. In addition, we found evidence that expression of miR-378a-3p was regulated by Hh/NF-κB signalling. Supplementary Fig. 13 shows that in qHSCs, the level of miR-378a-3p is high, so that miR-378a-3p blocks *gli3*, contributing to the lower expression of Gli3. When Smo is activated, it promotes the translocation of p65 into the nucleus. This activated p65 binds to the promoter (or control element) of the *miR-378a* gene, inhibiting its transcription. Reduced miR-378a-3p directly and indirectly increases Gli3 and Gli2, respectively, which accelerate the Hh signalling pathway and the expression of fibrotic genes, eventually promoting liver fibrosis (Supplementary Fig. 13). In addition, the data from the human HCC samples support the correlation between miR-378a-3p and *gli3*.

Several factors such as mouse strains, period of CCl_4_ administration and method of microarray analysis influence the outcomes of microarray analysis^30^,^31^, although the same chemical, CCl_4_, is used to induce liver fibrosis. We analysed liver tissues of C57BL/6 mice treated with CCl_4_ for 10 weeks using SurePrint G3 Mouse miRNA Microarray (Agilent), which covered 1,247 unique mouse miRNA targets using more advanced version (miRBase v19.0). Thus, genome-wide screening of miRNA expression using the more advanced version provided the updated miRNA expression profiles in liver fibrosis and revealed that expression of miR-378 family members including miR-378a-3p, miR-378b and miR-378d were the most significantly reduced among the differentially expressed miRNAs in CCl_4_-induced liver fibrosis, compared with control. This decreased expression of the miR-378 family was validated in fibrotic livers of mouse models with hepatic injury and also in aHSCs. A previous study also reported that the miR-378 significantly declined in liver tissues of rat with dimethynitrosamine (DMN)-induced hepatic fibrosis^32^.

The miRNA target prediction database (microRNA.org) predicted that the miR-378 family targets human *gli3* and *gli2* mRNA and mouse *gli3* and *gli2* mRNA. However, our luciferase reporter assay showed that only miR-378a-3p, and not miR-378b or miR-378d, bound directly to the 3′-UTR region of *gli3* mRNA in mouse (Fig. 3 and Supplementary Fig. 4). Mouse miR-378b and miR-378d have a similar sequence and structure to mouse miR-378a-3p, but a different seed sequence. As members of the same miRNA family with different seed sequences bind to different targets^33^, it seems that miR-378b and miR-378d do not bind to the 3′-UTR of *gli3* and *gli2* mRNA. Although neither miR-378b nor miR-378d binds to *gli2* and *gli3*, expression of both was changed in the injured liver, suggesting that they might have specific roles in the liver. Thus, further study is needed to investigate the functions of these two miRNAs in the liver. In the present study, we focused on the action of miR-378a-3p associated with Hh signalling.

HSCs are activated during HSC culture^20^,^21^,^34^. Hh signalling is one of the pathways stimulating the activation and proliferation of HSCs in an autocrine and paracrine manner^10^,^11^,^12^,^16^. The expression level of Hh signalling is very low in the qHSCs and starts to increase during activation of HSCs^20^,^35^. In addition, LSECs produce and respond to Hh ligands^36^,^37^,^38^, similar to HSCs. However, mature hepatocytes are not Hh-responsive cells, because they do not express Hh target genes such as *smo* and *Glis*^35^. In line with these results, our data showed that members of miR-378 were upregulated in the qHSCs and its' expression in these cells was similar with it in hepatocytes. aHSCs that are positive for Hh signalling showed the decreased expression of these miRNAs. Expression of miR-378 was also reduced in Hh-responsive LSECs, compared with both of the qHSCs and the hepatocytes that express lower Hh signalling (Fig. 2). Those findings suggest that the expression level of miR-378 also influences the expression of Hh signalling in hepatocytes and LSECs, including HSCs, by regulating Gli3 expression.

Our results demonstrate that g*li3* is a direct target of miR-378a-3p, as shown by bioinformatical analysis and luciferase reporter assay (Fig. 3). Although *Gli2*, another Hh target gene, was predicted to be a potential target of miR-378a-3p, no binding interaction between *gli2* and miR-378a-3p was observed. However, exogenous expression of miR-378a-3p reduced the expression of Gli3 and Gli2 in LX2 cells, suggesting that downregulation of Gli3 by miR-378a-3p did have an effect on the expression of Gli2, which is in agreement with previous reports that Glis mutually influence each other's expression^39^,^40^. In addition, g*li1* mRNA was downregulated in injured livers compared with normal livers (Supplementary Fig. 5a). In our present study, expression pattern of *gli1* mRNA was opposite to the expression of *gli2* and *gli3* mRNA, which indicated distinct functions of these transcriptional activators (Supplementary Fig. 5a–c). In line with our findings, Pritchett *et al*.^23^ demonstrated that *gli1* was rarely detected in qHSCs and unaltered during activation of HSCs, whereas expression of Gli2 and Gli3 was greatly higher in the aHSCs. In addition, we previously reported a lower expression of *gli1* mRNA in livers of rats with fibrosis^41^. In this previous research, we showed that miR-324-5p and miR-326 targeting *gli1* mRNA were highly expressed in activated LX2 cells. These results suggest that Gli2 and Gli3 function significantly in liver fibrogenesis.

NF-κB signalling regulates expression of genes responsible for immune responses and cell survival^42^. Among the NF-κBs composed of reticuloendotheliosis (Rel) proteins, including p50, p52, RelA (p65), RelB and c-Rel, which form homodimers or heterodimers, the p50-RelA (also known as p50–p65) heterodimer is the most abundant form of NF-κB^43^. Classically, NF-κB is sequestered in cytoplasm by inhibitors of κB (IκB), but phosphorylation of IκB by IκB kinase allows NF-κB to translocate into the nucleus and act as a transcription factor^42^. Recent studies have demonstrated that NF-κB regulates expression of miRNAs^44^,^45^. Roderburg *et al*.^44^ reported that NF-κB-dependent downregulation of the miR-29 family promoted the production of ECM proteins in HSCs. In the current study, we found evidence for a new concept that miR-378a-3p and NF-κB signalling are involved in activation of Hh signalling during liver fibrosis. Activation of NF-κB by Smo suppressed transcriptional expression of miR-378a-3p and the reduced miR-378a-3p caused an increase in Gli3 and Gli2, which in turn accelerated or promoted the Hh signalling and fibrotic response.

HCC is closely associated with liver fibrosis, as it frequently develops in areas of liver cirrhosis, which is the late stage of liver fibrosis^46^,^47^. Previous reports have shown that Hh signalling is strongly expressed in HCC tumour tissue^48^,^49^. aHSCs, the effector cells of liver fibrosis, promote the development of HCC through their immunomodulatory activities and ECM formation^50^,^51^,^52^. Recent studies report that miR-378a suppresses the growth of hepatitis B virus-related HCC by targeting the insulin-like growth factor 1 receptor^53^. A genetic variant in miR-378a DNA increases its promoter activity so that it acts as a tumour suppressor and leads to decreased susceptibility to HCC in the Chinese population^54^. We investigated expression differences of miR-378a-3p and *gli3* between tumour and non-tumour liver tissues from patients with HCC and severe fibrosis. We found that expression of miR-378a-3p decreased but expression of *gli3* mRNA increased in tumour compared with non-tumour tissues from each HCC sample, demonstrating the correlation of expressional regulation between miR-378a-3p and *gli3*. In addition, levels of the miR-378 family were also low in injured livers of mice fed for 3 or 4 weeks on an MCDE diet. In the MCDE-treated mouse model, the accumulated fatty hepatocytes undergo apoptosis and lead to increased inflammation, proliferation of progenitor cells and activation of HSCs, promoting the progression of liver disease to NASH with fibrosis^9^. Hh signalling is activated and contributes to this fibrogenic response of the liver to injury^7^,^8^. Hence, it is possible that the miR-378 family is involved in Hh signalling in the MCDE-treated mouse model, indicating that changes in expression of the miR-378 family are relevant to various types of chronic liver diseases. Taken together, our results indicate that miR-378a-3p could be a potential target for diagnosing and treating liver disease with activated Hh signalling.

A few recent studies reported that manipulating the expression of dysregulated miRNAs showed an anti-fibrotic effect in mouse model of CCl_4_-induced liver fibrosis^55^,^56^. However, the systemic delivery of RNA-based therapeutics *in vivo* remains a challenge due to the presence of serum nucleases in blood, the poor uptake by the cells in the targeted tissues and rapid clearance by the renal system^68^. An approach that can minimize these negative effects is nanomedicine. If properly engineered, viral-like features can be mimicked into the design of the NPs such as protection of genetic materials, cellular recognition, assist in the transport of genetic materials across cellular membranes and escape from endosome. In recent times, NPs made from an amino acid-based polymer incorporated with some of these abilities have been shown to effectively transfect tissue *in vivo*^57^,^58^. These NPs do not illicit the immune system due to the composition of the polymer and the surface decoration of polyethylene glycol (PEG), which has been shown to increase the circulation time in blood, and also have controlled release capabilities of nucleic acids in the cell's cytoplasm^58^,^59^. Thus, NPs fabricated with LTU2a, may be an ideal choice for miRNA delivery.

The *in vivo* results show the potential of NP/M to rescue liver induced with fibrosis even after a single injection. The control release of miR-378a-3p gradually normalizes the ALT and AST levels within 3 weeks after insults with CCl_4_, even though only 6 μg mg^−1^ of miR-378a-3p were released from the NPs after 1 week. During week 2 and 3, less than half the amount of miR-378a-3p was released but the persistence of release resulted in the gradual rise of the miR-378a-3p levels for each week post NP injection. Thus, the matching of the polymer degradation and release kinetics probably resulted in the normalization of the ALT and AST levels. Furthermore, the recovery of the liver function is observed by the normalization of Hh signalling, as evidence in the knockdown of Gli3, and matrix production, as shown by decreases in *α-sma*, *vimentin*, *col1α1* and *timp1* expressions. These results along with the histology, where the liver injected with NP/M shows similar morphology to the controlled tissues, directly substantiate our hypothesis for the mechanism of liver fibrosis and their potential therapy. Thus, these results merit further development of NPs fabricated with LTU2a as RNA delivery vehicles for nanomedicine.

In conclusion, our study demonstrates that miR-378a-3p suppresses the activation of HSCs by targeting *gli3*, and that Hh-responsive Smo represses the transcriptional expression of miR-378a-3p through p65, promoting the activation of Hh and profibrotic genes by increasing the expression of the Hh target genes, *Gli2* and *Gli3*, during liver fibrosis. These findings indicate that miR-378a-3p has great potential to be used as a biomarker of hepatic pathogenesis and a therapeutic agent for treating liver disease.

## Methods

### Experimental animal model

Male C57BL/6 mice were purchased from Hyochang (Dae-gu, Korea), housed with 12-h light/dark cycle and allowed free access to normal food and water. To induce liver fibrosis, 7-week-old mice received 0.6 ml kg^−1^ body weight of CCl_4_ (Sigma-Aldrich, St Louis, MO, USA) dissolved in corn oil by i.p. injection, twice a week for 6 (*n*=5) and 10 weeks (*n*=6)^44^. As a control, same number of mice was injected with equal volume of corn oil. All mice were killed, to obtain serum and liver sample, at 48 h post the last injection of CCl_4_ or corn oil. To examine the effect of miR-378 *in vivo*, 6-week-old mice received 0.4 ml kg^−1^ body weight of CCl_4_ (Sigma-Aldrich) dissolved in corn oil by i.p. injection, three times a week for 2 weeks^60^,^61^. Next, mice were randomly divided into three experimental groups, which were treated with 0.4 ml kg^−1^ body weight of CCl_4_, twice a weeks for 3 weeks in parallel with once i.p. injection of PBS (CCl_4_ group) or NP/M (6 nmol of miR-378a-3p mimic per mouse) or NP/NC (*n*=4 per group per week). As a control, the same number of mice was injected with equal volume of corn oil and NP/NC. Mice were killed to obtain serum and liver samples at 1, 2 and 3 weeks after injection of NPs or vehicle (Supplementary Fig. 12a). To induce NASH or control chow, 10-week-old mice were fed MCDE for 3 or 4 weeks (*n*=5 per group). Animal experiments with Hh inhibitor GDC-0449 (Selleck Chemicals, Houston, TX, USA) were described in the previous research^27^. Briefly, eight mice were treated with dimethylsulfoxide (DMSO; *n*=4) or 25 mg kg^−1^ GDC-0449 (*n*=4), to assess the effect of GDC-0449 in the healthy liver. Based on the previous study^62^, we determined the dose of GDC-0449. In the radiation model, GDC-0449 (*n*=5) or DMSO (*n*=4) was i.p. injected into mice 2 h before getting radiation. After irradiation, mice were given a daily dose of 25 mg kg^−1^ GDC-0449 or DMSO for 6 weeks and then killed. All animal care and surgical procedures were approved by the Pusan National University Institutional Animal Care and Use Committee and were carried out according to the provisions of the National Institutes of Health (NIH) guidelines for the Care and Use of Laboratory Animals.

### Human liver sample

Human HCC tissues with hepatitis B were provided by National Biobank of Korea (PNUH, Pusan, Korea (*n*=18)). Samples were graded for the stage 4 of fibrosis (F4), except one sample (F3). The paired tumour or non-tumour tissues were surgically resected from livers with HCC and frozen at −70 °C. The employed samples in these studies were 14 from male and 4 from female patients. In addition, 11 and 7 patients were non-alcohol and alcohol users, respectively. All samples derived from the National Biobank of Korea were obtained with informed consent under institutional review board-approved protocols (PNU IRB/2013_44_BR) and were studied in accordance with the NIH guidelines for human subject research.

### miRNA microarray

Total RNA including miRNA was isolated with TRIzol Reagent (Ambion, Life Technologies, Carlsbad, CA, USA) from liver tissues treated with CCl_4_ or corn-oil for 10 weeks, following the manufacturer's instructions. RNA quantity and quality were determined using a nanodrop (Thermo Scientific, Waltham, MA, USA) and Bioanalyzer (Agilent Inc., Santa Clara, CA, USA). Total RNA (100 ng) was labelled with desalted Cy3 using an miRNA Complete Labeling and Hyb Kit (Agilent) and then hybridized to an Agilent miRNA expression microarray in accordance with the manufacturer's instructions. A SurePrint G3 Mouse miRNA Microarray (Release 19.0, 8 × 60K; Agilent) platform was used for miRNA profiling and raw data were extracted using Feature Extraction Software (v11.0.1.1; Agilent). For microarray analysis, raw data were first filtered by a flag signal detected in all samples. Filtered raw data were processed using the Limma Bioconductor package (http://www.bioconductor.org/) in the R statistical environment (http://www.r-project.org/). After quantile normalization of data, miRNAs with twofold or greater differential expression were identified, with *P*-values of <0.05 being considered statistically significant.

### Cloning of vector constructs

Target genes of human and mouse miR-378 were predicted by bioinformatic analysis using the online database http://www.microrna.org/. Genomic DNA (gDNA) was isolated from normal mouse liver and its concentration and purity examined using Nanodrop (Thermo Scientific). The 3′-UTR of mouse *gli3* and *gli2*, containing binding sites for mouse miR-378, were amplified by PCR using mouse gDNA. The PCR product was purified using AccuPrep PCR Purification Kit (Bioneer, Daedeok-gu, Daejeon, Korea), cut by the restriction enzymes Xho1 and Not1, and then cloned into the psiCHECK-2 vector (Promega, Madison, WI, USA). The vector constructs with 3′-UTR of *gli3* and *gli2* were transformed into *Escherichia coli* and then plasmid DNA was extracted from well-transformed, ampicillin-resistant *E. coli*, using an AccuPrep Plasmid Mini Extraction Kit (Bioneer). The sequences of the miR-378-binding sites of the 3′-UTR of *gli3* and *gli2* were confirmed by sequencing analysis (Macrogen, Seoul, Korea). Mutant vectors lacking the miR-378-binding site were produced by site-directed mutagenesis using a QuikChange Site-Directed Mutagenesis Kit (Stratagene, Agilent) in accordance with the manufacturer's instructions. All primer sequences used for vector construction are listed in Table 1.

Putative binding sites of transcription factors were analysed by TRANSFAC database (assembly Grch37/hg19). According to a database of microRNA TSS (miRStart), transcription start site is resided at −1,069 nucleotides (nt) on the *miR-378a* gene (chr5:149112388–149112453). Promoter region from −961 to −190 nt including binding site of vertebrate p65 (+p65BS) were amplified by PCR using human gDNA. In addition, promoter regions from −752 to −190 nt excluding p65-binding site were amplified by the same system (+/Δp65BS). Purified PCR products by AccuPrep PCR Purification Kit (Bioneer) were cut by the restriction enzymes Nhe1 and Xho1, and inserted into the pGL3-basic vector (Promega). The vector constructs with promoter region of primary (pri)-miR-378a were transformed into *E. coli* and then plasmid DNA was extracted from well-transformed and ampicillin-resistant *E. coli* using an AccuPrep Plasmid Mini Extraction Kit (Bioneer). The inserted regions in these experiments were confirmed by sequencing analysis (Macrogen). All primer sequences used for vector construction are listed in Table 1.

### Isolation of primary liver cells

Primary hepatic cells including hepatocytes, HSCs and LSECs were isolated as described previously^63^,^64^,^65^,^66^. Briefly, male C57BL/6 mice weighing 26–30 g were anaesthetized with zoletil 50 (5 mg kg^−1^ body weight, Virbac SA, France), to immobilize in the recumbent position on a treatment table, and the inferior vena cava was cannulated under aseptic conditions. Livers were perfused *in situ* with EGTA and collagenase (Roche, Indianapolis, IN, USA), to disperse the cells. Primary hepatocytes were separated from non-parenchymal cells using Percoll density gradient centrifugation. Isolated hepatocytes were cultured in DMEM medium (Gibco, Life Technologies, Carlsbad, CA, USA) containing 10% fetal bovine serum (FBS, Gibco, Life Technologies) and 1% penicillin/streptomycin (P/S, Gibco, Life Technologies). Primary HSCs and LSECs were isolated by differential centrifugation on OptiPrep (Sigma-Aldrich) density gradient and located on the upper layer of 11.5% and 20% OptiPrep, respectively. The purity of HSCs was >98%, as established by microscopy examination for lipid droplets and vitamin A autofluorescence. The upper layer of 20% OptiPrep contains LSEC and Kupffer cell fraction. As LSECs poorly attach culture plastic dish, LSECs are separated from Kupffer cell fraction by selective adherence. Isolated HSCs and LSECs were cultured in RPMI 1640 medium (Gibco, Life Technologies) containing 10% FBS and 1% P/S at 37 °C in a humidified atmosphere containing 5% CO_2_. As determined by trypan blue exclusion, cell viability was >92% in all experiments.

### Cell experiments

LX2 cell, human HSC line (provided by Dr Jeong, KAIST, KOREA), HepG2 cell, human hepatocyte cell line (provided by Dr Jang, PNU, KOREA) and N2a cell, mouse neuroblastoma cell line (provided by Dr Youn, PNU, KOREA), were cultured in DMEM supplemented with 10% FBS and 1% P/S at 37 °C in a humidified atmosphere containing 5% CO_2_. To evaluate the effect of miR-378 on HSC activation, LX2 cells (1.5 × 10^5^ per well) cultured for 24 h were transfected with 20 nM of miR-378 mimic (AccuTarget human miRNA-378a-3p mimic, Bioneer) or 20 nM of scrambled miRNA (miRNA mimic negative control 1, Bioneer) as a negative control using Lipofectamine RNAi/MAX transfection reagent (Invitrogen, Life Technologies, Carlsbad, CA, USA), according to the manufacturer's instructions. After 6 h, the medium was changed with fresh medium containing 2% FBS and 1% P/S, and then these transfected cells were incubated at 37 °C and 5% CO_2_ atmosphere for 24 or 48 h. To inhibit Smo, fully activated LX2 cells were treated with Vismodegib (1 μM of GDC-0449; Selleck Chemicals), Smo antagonist or vehicle (DMSO) for 24 and 48 h. To assess the regulation of pri-miR-378a expression by NF-κB, HepG2 cells were treated with TNF-α (100 ng ml^−1^), activator of NF-κB signalling or vehicle (PBS), and LX2 and HepG2 cells were treated with Bay 11-7085 (2 μM), repressor of NF-κB or vehicle (DMSO) for 24 h. To evaluate the relationship of Smo with NF-κB, LX2 and HepG2 cells were treated with GDC-0449 (1 μM) for 18 h, followed by addition of TNF-α (100 ng ml^−1^). These cells were harvested at 6 h post treatment of TNF-α. As determined by trypan blue exclusion, cell viability was >90% in all experiments. The data were shown as the mean±s.e.m. obtained from three different experiments.

### Luciferase reporter assay

One day before transfection, 10^6^ N2a or HepG2 cells were spread over 24-well culture plates in culture medium without antibiotics. Using Lipofectamine 2000 (Invitrogen), N2a cells were transfected with a mixture of psiCHECK-2 vector construct and either an miR-378a-3p mimic (25 nM; AccuTarget mouse miRNA-378a-3p mimic; Bioneer) or the same concentration of scrambled miRNA (miRNA mimic negative control 1; Bioneer) as a negative control. HepG2 cells were transfected with a mixture of pGL3-vector construct and CDH-p65 or CDH-GFP (control) using Lipofectamine 2000. CDH-p65 was a CDH-GFP lentiviral vector cloned by p65 construct^67^. At 24 h after transfection, cells were harvested and tested with the Dual Luciferase Reporter Assay System (Promega) in accordance with the manufacturer's protocol. All firefly luciferase activity data are normalized to *Renilla* luciferase activity and presented as the mean±s.e.m. of values from at least three repetitive experiments.

### RNA analysis

Total RNA was extracted from liver tissues or cells by using TRIzol reagent (Ambion, Life Technologies) or miRNeasy Mini Kit (Qiagen, Valencia, CA, USA) to enrich miRNA quantity. The concentration and purity of RNA were determined using a nanodrop. Template complementary DNA was synthesized from total RNA using the SuperScript First-strand Synthesis System (Invitrogen, Life Technologies) or miScript Reverse Transcriptase Kit (Qiagen) according to the manufacturer's protocols. We performed the real-time qRT–PCR analysis by using Power SYBR Green Master Mix (Applied Biosystem, Life technologies) or miScript SYBR Green PCR Kit (Qiagen) on the manufacturer's specifications (Eppendorf, Mastercycler Real-Time PCR). All reactions were triplicated and data were analysed according to the ΔΔC_t_ method. 40S ribosomal protein S9 mRNA and 18S ribosomal RNA for mRNA and U1A small nuclear RNA (RNU1A) for miRNA were used for normalization of the expression level. In data analysis for the expression of miR-378a-3p and *gli3* in human samples, average of expression of miR-378a-3p or *gli3* from all paired non-tumour and tumour tissues was calculated. Next, individual value of expression relative to the mean value was presented as scatter plot and mean±s.e.m. of each group was graphed. The sequences of all primers used in this study are summarized in Supplementary Tables 1 and 2. All PCR products were directly sequenced for genetic confirmation (Macrogen).

### Western blotting

Total protein was extracted from frozen liver tissues stored at −80 °C and cultured cells. Samples were homogenized in triton lysis buffer supplemented with protease inhibitors (Roche) and centrifuged at 13,000 r.c.f. for 15 min. The supernatants containing protein extracts were used in subsequent biochemical analysis. Protein concentration was measured by Pierce BCA Protein Assay Kit (Thermo Scientific). Equal amount of total protein lysates were separated by 8 or 10% SDS–PAGE and then transferred onto a polyvinylidene difluoride membrane (Millipore, Darmstadt, Germany). Primary antibodies used in this study were as follows: rabbit anti-Smo antibody (diluted 1:1,000; Abcam), rabbit anti-Gli2 antibody (diluted 1:1,000; GenWay Biotech, Inc., San Diego, CA, USA), rabbit anti-Gli3 antibody (diluted 1:1,000; Abcam), rabbit anti-transforming growth factor-β antibody (diluted 1:1,000; Cell Signaling Technology, Inc., Danvers, MA, USA), mouse anti-α-SMA antibody (diluted 1:1,000; Sigma-Aldrich), rabbit anti-p65 antibody (diluted 1:1,000; Santa Cruz Biotechnology, Inc., Santa Cruz, CA, USA), rabbit anti-GFAP antibody (diluted 1:1,000; Dako, Carpinteria, CA, USA) and mouse anti-glyceraldehydes 3-phosphate dehydrogenase antibody (diluted 1:1,000; AbD Serotec, Oxford, UK) as an internal control. Horseradish peroxidase-conjugated anti-rabbit or anti-mouse IgG (Amersham ECL, GE Healthcare, Milwaukee, WI, USA) was used as secondary antibody. Protein bands were detected using an EzWestLumi ECL solution (ATTO Corporation, Tokyo, Japan) as per the manufacturer's specifications (ATTO Corporation, Ez-Capture II). Densities of protein bands were measured using CS Analyzer software (Version 3.00.1011, ATTO & Rise Corporation).

To separate nuclear and cytosolic fraction from total protein, harvested cells were resuspended with buffer A (10 mM HEPES, 50 mM NaCl, 1 mM dithiothreitol, 0.1 mM EDTA and 0.1 mM phenylmethylsulfonyl fluoride) and incubated on ice for 20 min. After adding 0.1% of NP-40, lysated cells were incubated for 20 min additionally. After centrifugation at 5,000 *g* for 2 min, the supernatant was collected for cytosolic fraction. Pellet was resuspended with buffer B (20 mM HEPES, 400 mM NaCl, 1 mM dithiothreitol, 1 mM EDTA, 1 mM phenylmethylsulfonyl fluoride and 1 mM EGTA) and incubated on ice for 30 min. After centrifugation at 13,000 r.p.m. for 15 min, the supernatant was saved for nuclear fraction. Rabbit-anti-Lamin β1 antibody (diluted 1:1,000; Abcam) was used as a marker of nuclear protein and internal control.

### Liver histology and immunohistochemical stain

To examine hepatic morphology and assess liver fibrosis, H&E staining and Sirius red staining were performed, respectively. Liver specimens were fixed in 10% neutral buffered formalin (Sigma), embedded in paraffin and cut into 4 μm sections. Next, the specimens were deparaffinized, hydrated and stained by standard methods.

For immunohistochemistry, liver sections were deparaffinized, hydrated and incubated in 3% hydrogen peroxide, to block endogenous peroxidase. Antigen retrieval was performed by heating in 10 mM sodium citrate buffer (pH 6.0) for 10 min using microwave. Specimens were blocked in Protein Block solution (Dako) for 30 min at room temperature (RT) followed by incubation with primary antibody at 4 °C overnight. Other sections were also incubated at 4 °C overnight in non-immune sera. Rabbit α-SMA antibody (diluted 1:500; Abcam) was used as a primary antibody and diluted in Protein Diluent (Dako). Polymer-horseradish peroxidase anti-rabbit (Dako) was used as secondary antibody and 3,3′-diaminobenzidine as brown colour was used to visualize the protein.

### Hydroxyproline assay

Hydroxyproline content of the livers was calculated by the method previously described^9^,^41^. Briefly, 50 mg of freeze-dried liver tissue was hydrolysed in 6 N HCL at 110 °C for 16 h. The hydrolysate was evaporated under vacuum and the sediment was re-dissolved in 1 ml distilled water. Samples were filtered using 0.22 μm filter centrifuge tube at 14,000 r.p.m. for 5 min. Lysates were then incubated with 0.5 ml of chloramines-T solution containing 1.41 g of chloramine-T dissolved in 80 ml of acetate–citrate buffer and 20 ml of 50% isopropanol, at RT. After 20 min, 0.5 ml of Ehrlich's solution, containing 7.5 g of dimethylaminobenzaldehyde dissolved in 13 ml of 60% perchloric acid and 30 ml of isopropanol, was added to the mixture, which was incubated at 65 °C for 15 min. After cooling to the RT, the absorbance was read at 561 nm. Amount of hydroxyproline in each sample was determined using the regression curve from the hydroxyproline prepared with high purity hydroxyproline (Sigma-Aldrich) and divided by the amount of liver weight contained in the initial sample (50 mg) to get the hydroxyproline contents (μg hydroxyproline per mg liver). Data were expressed as fold changes by comparing with hydroxyproline content of the control group.

### Measurement of ALT and AST

The levels of serum ALT (GPT, glutamate–pyruvate transaminase) and AST (GOT, glutamate–oxaloacetate transaminase) were measured using GOT·GPT measuring reagents (Asan Pharmaceutical, Seoul, Korea) according to the manufacturer's instructions.

### Polymer synthesis

A ‘pseudo' poly(amino acid)-based polyurethane has been synthesized according to previous established protocol^58^,^68^. The synthesis of the LTU2a was completed first by the formation of hexyl ester of L-tyrosine. Next, desaminotyrosyl-tyrosine-hexyl ester was synthesized by coupling hexyl ester of L-tyrosine to desamino tyrosine (Sigma-Aldrich) through a carbodiimide reaction. Finally, a pre-polymer of PEG (molecular weight 2,000 Da, Sigma) and hexamethylene diisocynate (Sigma-Aldrich) was formed using stannous octoate (Sigma-Aldrich). The resulting polymer was precipitated in a chilled sodium chloride solution (12 g l^−1^). The product was vacuum dried at 40 °C for 3 days before characterization. The LTU2a synthesis yielded a polymer with an average molecular weight of 36,270 Da and polydispersity index of 1.6. Once the product has been verified with nuclear magnetic resonance (NMR) and gel permeation chromatography (GPC), LTU2a was used to make NPs.

### NP formulation

NPs were formulated by blending LTU2a (197 mg dissolved in 9 ml of chloroform), copolymer of PEG-PLA (2 mg dissolved in 1 ml of chloroform) and linear polyethylenimine (LPEI) complexed with miRNA (100 nmol of miR-378a-3p or 100 nmol of scrambled miRNA at mass ratios of 5:1) or 2 mg of FITC–BSA (Sigma-Aldrich) dissolved in 1 ml of diethylpyrocarbonate (DEPC)-treated water using a water-in-oil-in-water emulsion and solvent evaporation^57^,^58^. The initial emulsion was formed with a high-speed mixer at 2,000 r.p.m. in a round-bottom glass flask for 3 min. Afterwards, the speed was lowered to 1,600 r.p.m., 5% polyvinyl alcohol (PVA) (100 ml, Sigma-Aldrich) was added and mixed for an additional minute. The organic solvent was allowed to evaporate overnight. The NPs were washed by centrifugation and lyophilized for 3 days.

### NP characterization

Five milligrams of either NP/M or NP/NC was suspended in 5 ml of distilled and deionized water. The 10 μl of NP solution was pipetted onto a copper grid. After water evaporation, transmission electron microscopy (JEOL 2010) images were obtained. Dynamic light scattering (DLS) analysis (Nano ZS, Malvern) and zeta potential (Nano ZS, Malvern) were obtained using 1 ml of the NP solution.

### miRNA release studies

Release studies of miRNA were performed according to published methods^57^,^59^. Five milligrams of NP/M or NP/NC was incubated with 1 ml of DEPC-treated water in a shaker bath set at 37 °C. The samples were centrifuged, the supernatant was collected and the NPs were resuspended at days 1, 2, 4, 7, 14, 21, 28, 35 and 42. The amount of release for miR-378a-3p and scrambled miRNA was determined with Quant-iT PicoGreen assay (Thermo Scientific). Known standards of miRNA–LPEI complexes were used to calculate the release profile of LTU2a-NPs^57^,^58^.

### Uptake studies

LX2 cells were seeded onto German coverslips at 1.5 × 10^4^ cells per cm^2^ and cultured as previously described. Next day, NPs encapsulated with FITC–BSA were suspended in cell culture medium at 0.1 mg ml^−1^ and exposed to cells for ∼24 h. Next, the cells were fixed with 0.1% formaldehyde solution in PBS. As an optional procedure, the fixed cells were stained rhodamine phalloidin (Thermo Scientific). The coverslips were mounted with an anti-fade reagent (VectorShield) and imaged with fluorescence (Nikon) or confocal microscopy (Zeiss).

### Statistical analysis

Results are expressed as the mean±s.e.m. Statistical significances between control and treated groups or subgroups were analysed by the unpaired two-sample Student's *t*-test or one-way analysis of variance followed by a *post-hoc* Tukey's test. Data for *in vivo* effect of miR-378 in the liver were first analysed with the non-parametric Kruskal–Wallis test and then the differences between subgroups were further analysed by the two-sample Student's *t*-test. Differences were considered as significant when *P*-values are <0.05. Results from HCC patients were expressed as individual values of relative gene expression and the mean±s.e.m., to show distributions of most variables in patients. Difference between non-tumour and tumour group was determined using the paired two-sample Student's *t*-test and considered as significant when *P*-values are <0.05. The degree of correlation between the expression levels of miR-378a-3p and *gli3* in liver tissues of HCC and between the level of miR-378a-3p and hydroxyproline content or Gli3 in liver tissues of CCl_4_-treated mice were analysed by the Spearman's rank correlation coefficient (*ρ*). Statistical analyses were performed using IBM SPSS Statistics 21 software (Release version 21.0.0.0, IBM Corp., Armonk, NY, USA).

## Additional information

**Accession codes:** The microarray data have been deposited in NCBI GEO database under the accession code GSE77271.

**How to cite this article:** Hyun, J. *et al*. MicroRNA-378 limits activation of hepatic stellate cells and liver fibrosis by suppressing Gli3 expression. *Nat. Commun.* 7:10993 doi: 10.1038/ncomms10993 (2016).

## Supplementary Material

Supplementary Information Supplementary Figures 1-14 and Supplementary Tables 1 – 2.

## Figures and Tables

**Table 1:** Primer sequences used for cloning.

**Gene**	**Primer sequences (5′–3′)**
gli2 wt 3′UTR_F	TTTTCTCGAGTCTCTGGCTCTTGTGGTGTG
gli2 wt 3′UTR_R	TTGCGGCCGCCACCAAATTTACTGCCTGGA
gli3 wt 3′UTR_F	TTTTCTCGAGGAGTCAAAAGTGTTCTATCCCAAGA
gli3 wt 3′UTR_R	TTGCGGCCGCTTCCTGGTGACACTGTCTGG
gli2 mut 3′UTR_F	GTTTGGCAATATAAATTTGGGTCGTCCAAATTTGGATTCACCCACAGTC
gli2 mut 3′UTR_R	GACTGTGGGTGAATCCAAATTTGGACGACCCAAATTTATATTGCCAAAC
gli3 mut 3′UTR-1_F	CACCAATCTGTCCTGATTGACGACTTCAAGAATGATCATTTGG
gli3 mut 3′UTR-1_R	CCAAATGATCATTCTTGAAGTCGTCAATCAGGACAGATTGGTG
gli3 mut 3′UTR-2_F	CAGCCTCTGAGTGCTGACTCCTCAACAACAACAACTGGTCC
gli3 mut 3′UTR-2_R	GGACCAGTTGTTGTTGTTGAGGAGTCAGCACTCAGAGGCTG
+p65 BS_F	TTTGCTAGCCACTTGCTGCCGTACTTTCA
+p65 BS_R	TTTTCTCGAGCTCCCACTCCAGGTACCAAA
Δp65 BS_F	TTTGCTAGCGACAGCGTCTTCCTGGTTTC

F, forward; mut, mutant; R, reverse; wt, wild-type.

Primer sequences shown in this table were used for cloning the vector constructs.
