# Supplementary material for: MicroRNA-378 limits activation of hepatic stellate cells and liver fibrosis by suppressing Gli3 expression
Source: Nat Commun. 2016 Mar 22;7:10993. doi: 10.1038/ncomms10993 (PMC4804167; doi:10.1038/ncomms10993)
Supplement: Supplementary Information — Supplementary Figures 1-14 and Supplementary Tables 1 – 2. [file ncomms10993-s1.pdf]

Supplementary Figures

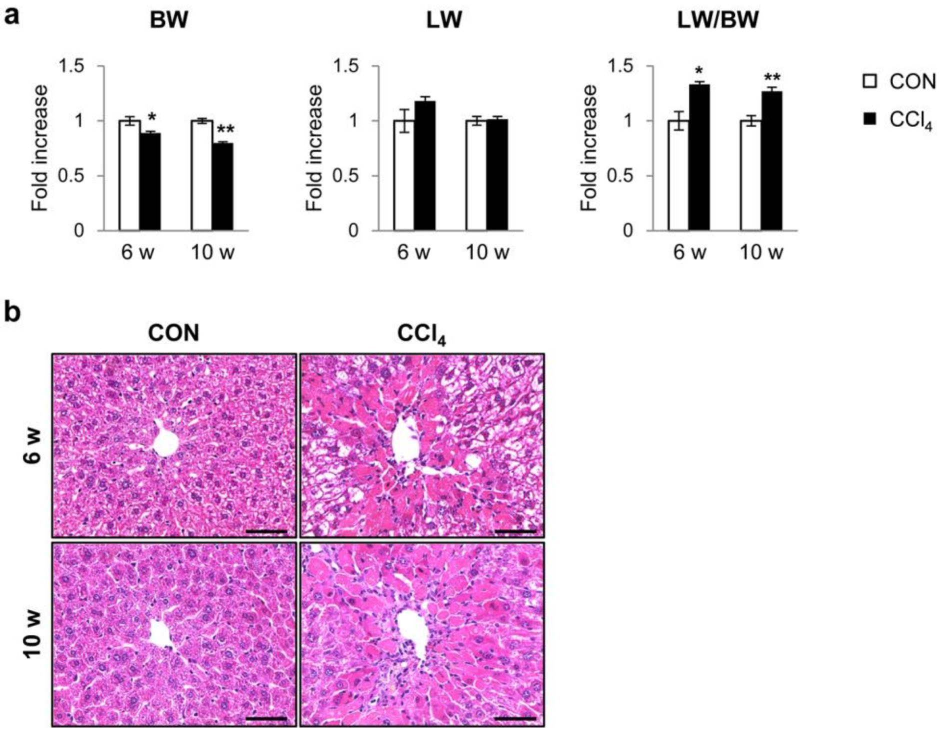

Supplementary Figure 1. CCl_4 injures liver of mice. (**a**) Body weight (BW), liver weight (LW), and the ratio of liver to body weight (LW/BW) of male C57BL/6 mice treated with CCl_4 (0.6 ml kg^{-1} BW, twice a week, i.p.) or corn-oil for 6 (total n=10) or 10 (total n=12) weeks. Results were graphed as mean±s.e.m. (unpaired two-sample Student's t-test, *$p<0.05$, **$p<0.005$ vs. CON). (**b**) Liver morphology was assessed by hematoxylin and eosin (H&E) staining for liver sections from representative CON and CCl_4-treated mice (scale bar = 50 µm).

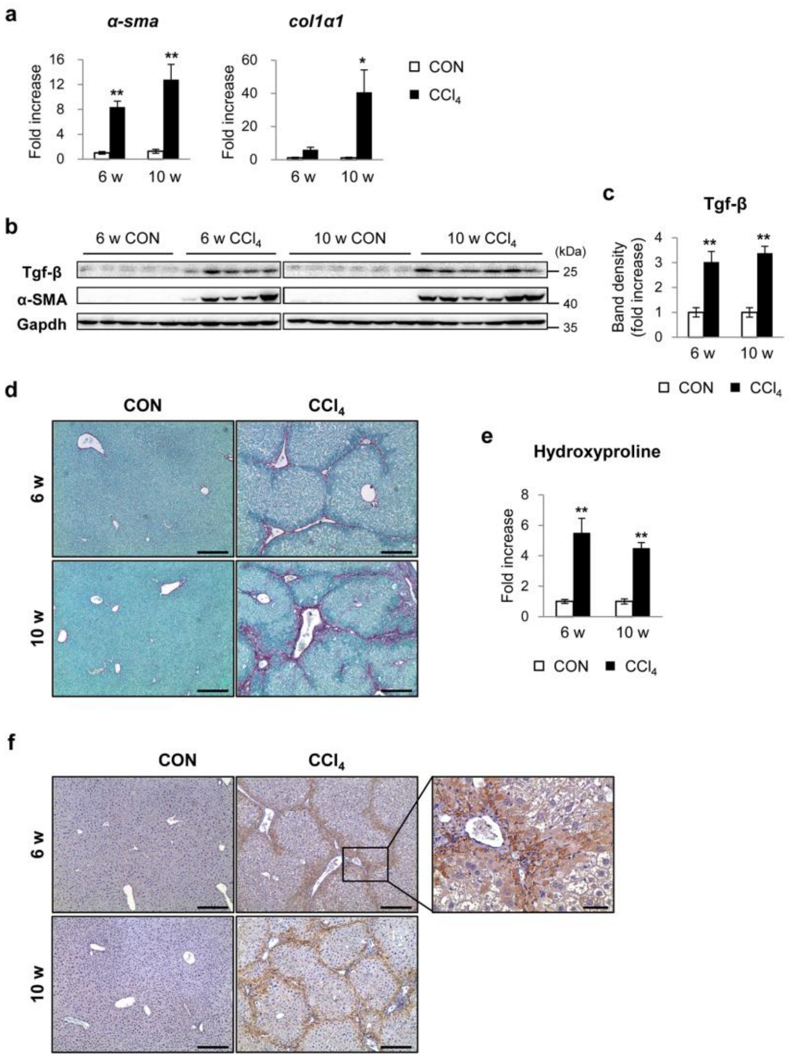

Supplementary Figure 2. CCl_4-induced liver fibrosis in mice. (a) qRT-PCR of *α-sma* and *col1α1* in livers from corn-oil (CON) or CCl_4-injected mice for 6 or 10 weeks (n=4/group). (**b**) Western blot analysis and (**c**) cumulative densitometry analyses for Tgf-β (25 kDa) and α-SMA (42 kDa) in livers from representative CON and CCl_4-treated mice for 6 (CON; n=5, CCl_4; n=5) or 10 (CON; n=6, CCl_4; n=6) weeks. GAPDH was used as an internal control. Data shown represent one of three experiments with similar results. (**d**) Liver fibrosis was assessed by Sirius red staining for liver sections from representative CON and CCl_4-treated mice (scale bar = 100 µm). (**e**) Hepatic hydroxyproline content in all mice. (**f**) Immunohistochemistry for α-SMA in liver sections from representative CON and CCl_4-treated mice (scale bar = 100 µm). The stained cells are shown in the magnified images with detail (scale bar = 20 µm). All results are displayed as mean±s.e.m. and the unpaired two-sample Student's t-test was used for statistics (*p<0.05, **p<0.005 vs. CON).

a

miRNA	Fold change (CCl_4/CON)	Adj. *p*-value
miR-378a-5p	0.47444	0.18209

b

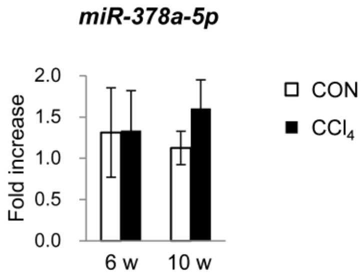

Supplementary Figure 3. Expression analysis of miR-378a-5p in livers of mice. (**a**) miR-378a-5p in CCl_4-treated compared with corn-oil-treated livers (CON) is shown, with the fold change and *p*-values. (**b**) qRT-PCR was performed to assess expression of miR-378a-5p in livers from the CON and CCl_4-treated mice at 6 and 10 weeks (n=4/group). Mean±s.e.m results are graphed (unpaired two-sample Student's t-test, *p<0.05 vs. CON).

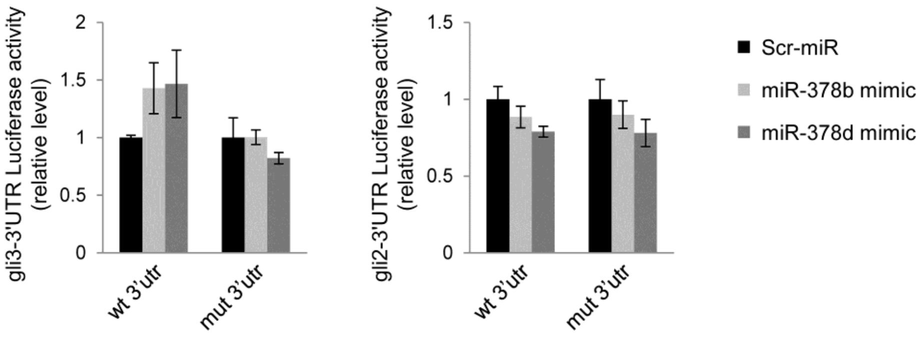

Supplementary Figure 4. Neither miR-378b nor miR-378d bind to *gli2* or *gli3* mRNA. Dual luciferase reporter assay was performed to examine whether miR-378b or miR-378d bound to *gli2* or *gli3* mRNA. N2a was co-transfected with psiCHECK-2 vectors containing the wild-type (wt) or mutant (mut) target sites and miR-378b or miR-378d mimic. Scrambled (Scr)-miR oligonucleotides was transfected as a control. Results of relative luciferase activity were shown as mean±s.e.m. obtained from three repetitive experiments. The unpaired two-sample Student's t-test was used for statistics.

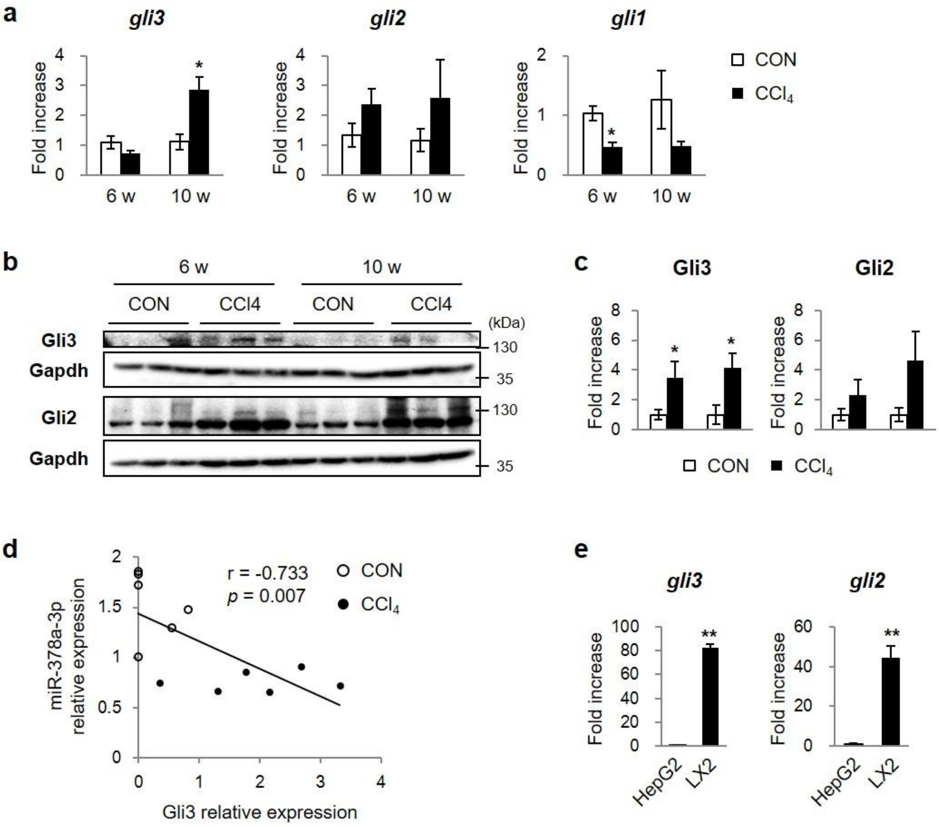

Supplementary Figure 5. Activation of Hh signaling in fibrotic livers of mice and activated HSC. (**a**) qRT-PCR of *gli3*, *gli2*, and *gli1* in livers from corn-oil (CON) or CCl_4-injected mice for 6 or 10 weeks (n=4/group). Mean±s.e.m. results are graphed (**p*<0.05 vs. CON). (**b**) Western blot analysis for Gli2 and Gli3 in livers from representative CON and CCl_4-treated mice (n=3/group). GAPDH was used as an internal control. Data shown represent one of three experiments with similar results. (**c**) Cumulative densitometric analyses of Gli3 and Gli2 western blots results are displayed as the mean±s.e.m. (**p*<0.05). (d) Spearman's rank correlation between miR-378a-3p expression and Gli3 protein expression in liver of mice (n=12, Spearman's rank correlation analysis; r, correlation coefficient). (**e**) qRT-PCR of g*li3* and g*li2* in LX2 and HepG2 cells. All results of relative expression values were shown as mean±s.e.m. of triplicate experiments (***p*<0.005 vs. HepG2). The unpaired two-sample Student's t-test was used for statistics.

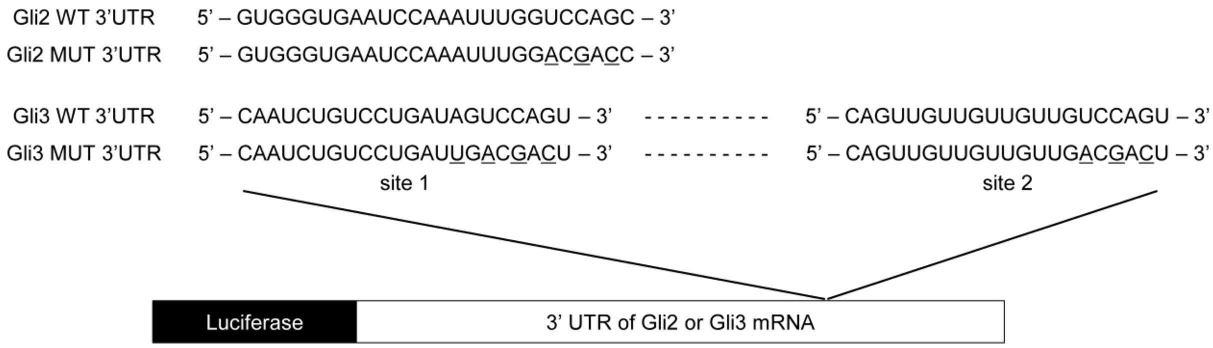

Supplementary Figure 6. psiCHECK-2 vectors having either wild-type or mutant binding site of miR-378a-3p in 3' UTR of *gli2* and *gli3* mRNA. The wild-type (WT) or mutated (MUT) nucleotides of miR-378a-3p binding site on 3' UTR of *gli2* and *gli3* mRNA were shown. The mutated nucleotides were underlined.

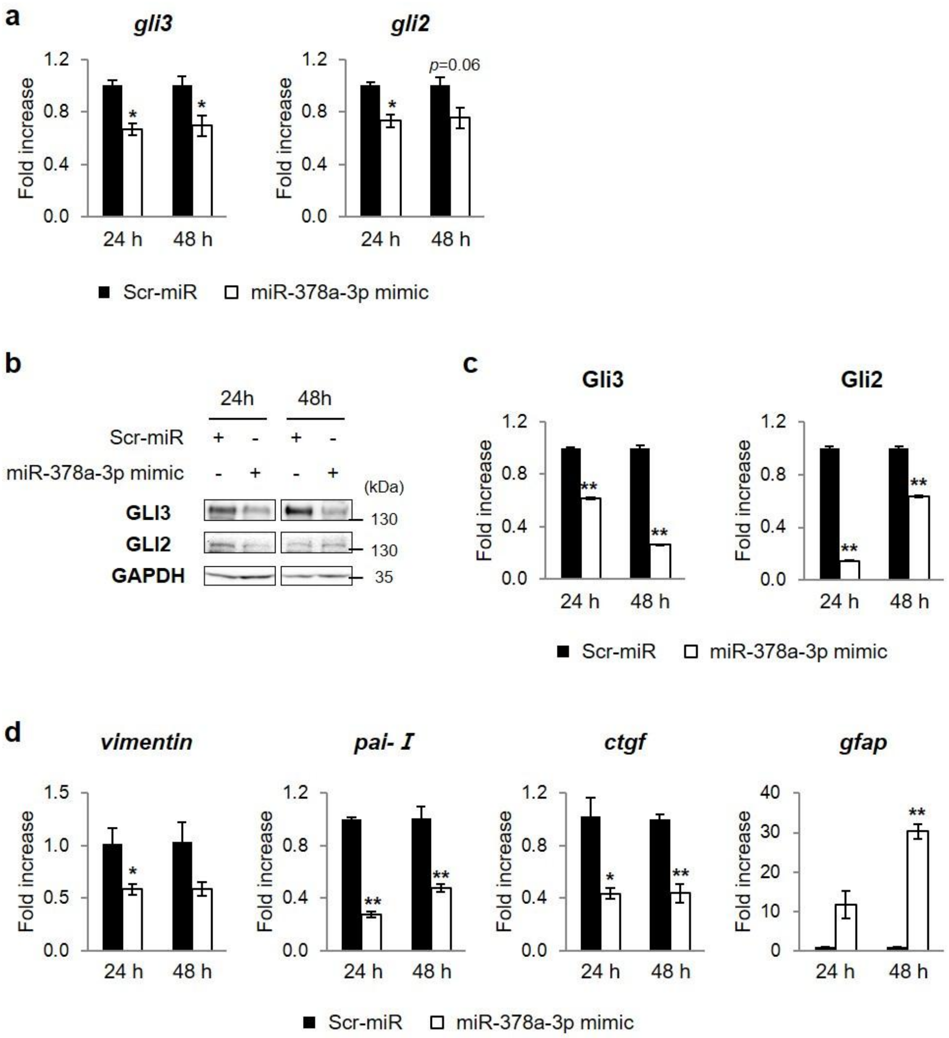

Supplementary Figure 7. Overexpression of miR-378a-3p in LX2 cells reduced expression of Gli3 and Gli2, inducing inactivation of HSC. (**a**) Activated LX2 cells were transfected with either an miR-378a-3p mimic (white bar) or scrambled (Scr)-miR (control) (black bar) oligonucleotide for 24 and 48 hours, and expression of *gli3* and *gli2* was assessed by qRT-PCR. (**b**) Western blot analysis and (**c**) cumulative densitometric analyses for Gli3 (170 kDa) and Gli2 (133 kDa), with GAPDH (36 kDa) as internal control. Data shown represent one of three experiments with similar results. (**d**) qRT-PCR analysis for genes related to activation of HSC, including *vimentin*, *pai-I*, *ctgf*, and the inactivation marker of HSC, *gfap*, in LX2 cells transfected with miR-378a-3p mimic (white bar) or scrambled (Scr)-miR (control) (black bar) oligonucleotide for 24 and 48 hours. All results of relative expression values are shown as mean±s.e.m. obtained from triplicate experiments (unpaired two-sample Student's t-test, * $p<0.05$, ** $p<0.005$ vs. Scr-miR).

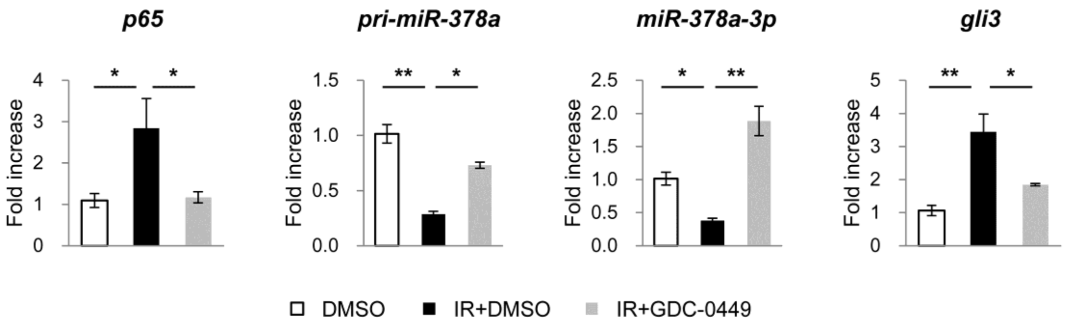

Supplementary Figure 8. Hh inhibitor, GDC-0449, results in up-regulation of miR-378a and down-regulation of *p65* and *gli3* in irradiated mice with liver fibrosis. (**a**) qRT-PCR of *p65*, *pri-miR-378a*, *miR-378a-3p*, and *gli3* in livers from vehicle (DMSO)-treated, irradiated mice with DMSO (IR+DMSO) or GDC-0449 (IR+GDC-0449) (n=5/group). Mean±s.e.m. results are graphed (one-way ANOVA with Tukey corrections, *p<0.05, **p<0.005 vs. DMSO).

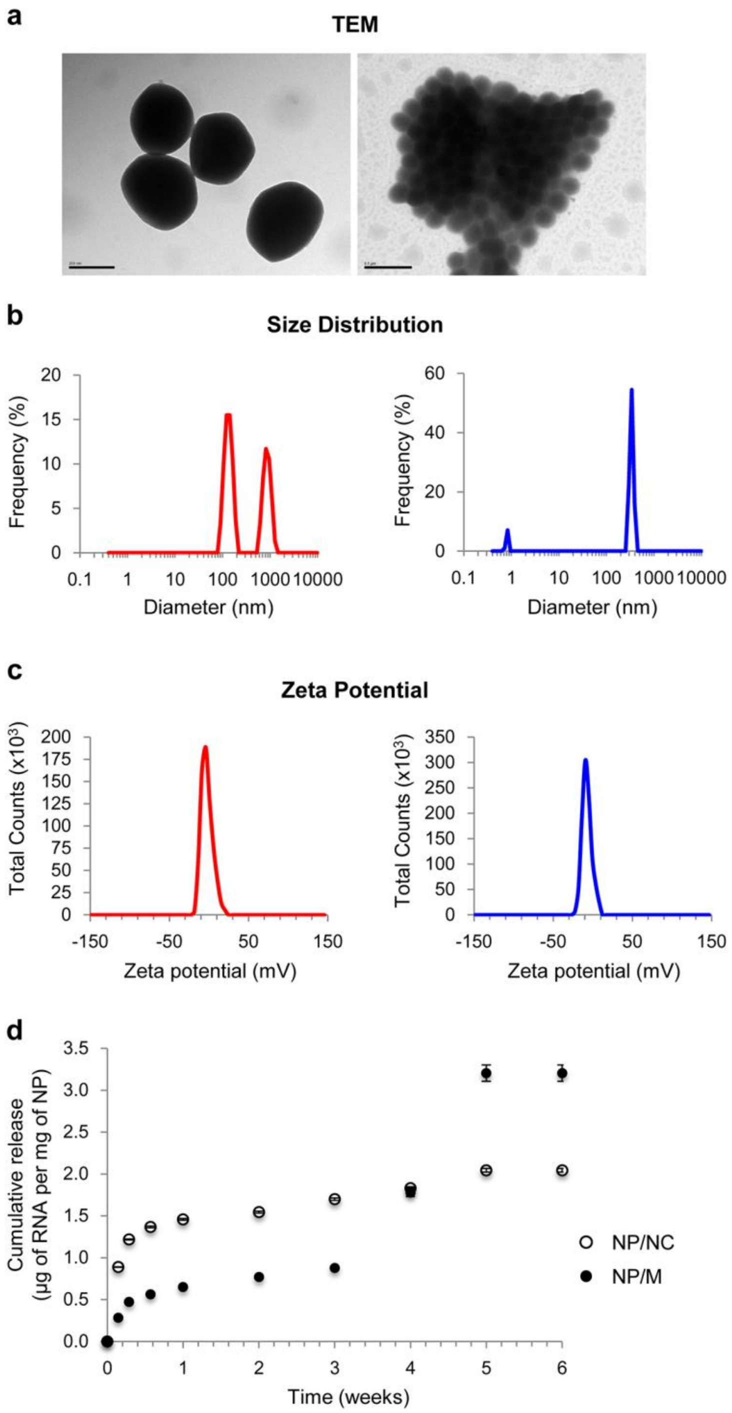

Supplementary Figure 9. Generation of LTU2a-NPs harboring miR-378a-3p mimic or scrambled miRNA. (**a**) Transmission electron microscopy (TEM) for LTU2a-NP/scr-miR (NP/NC) (left, scale bar = 200 nm) and LTU2a-NP/miR-378a-3p (NP/M) (right, scale bar = 0.5 µm) at an accelerating voltage of 200 kV. (**b**) Size distributions of NPs/NC (left) and NPs/M (right) by dynamic light scattering (DLS) analysis. (**c**) Zeta-potential of NPs/NC (left) and NPs/M (right) by DLS analysis. (**d**) In vitro release kinetics of miRNA releasing for both formulations.

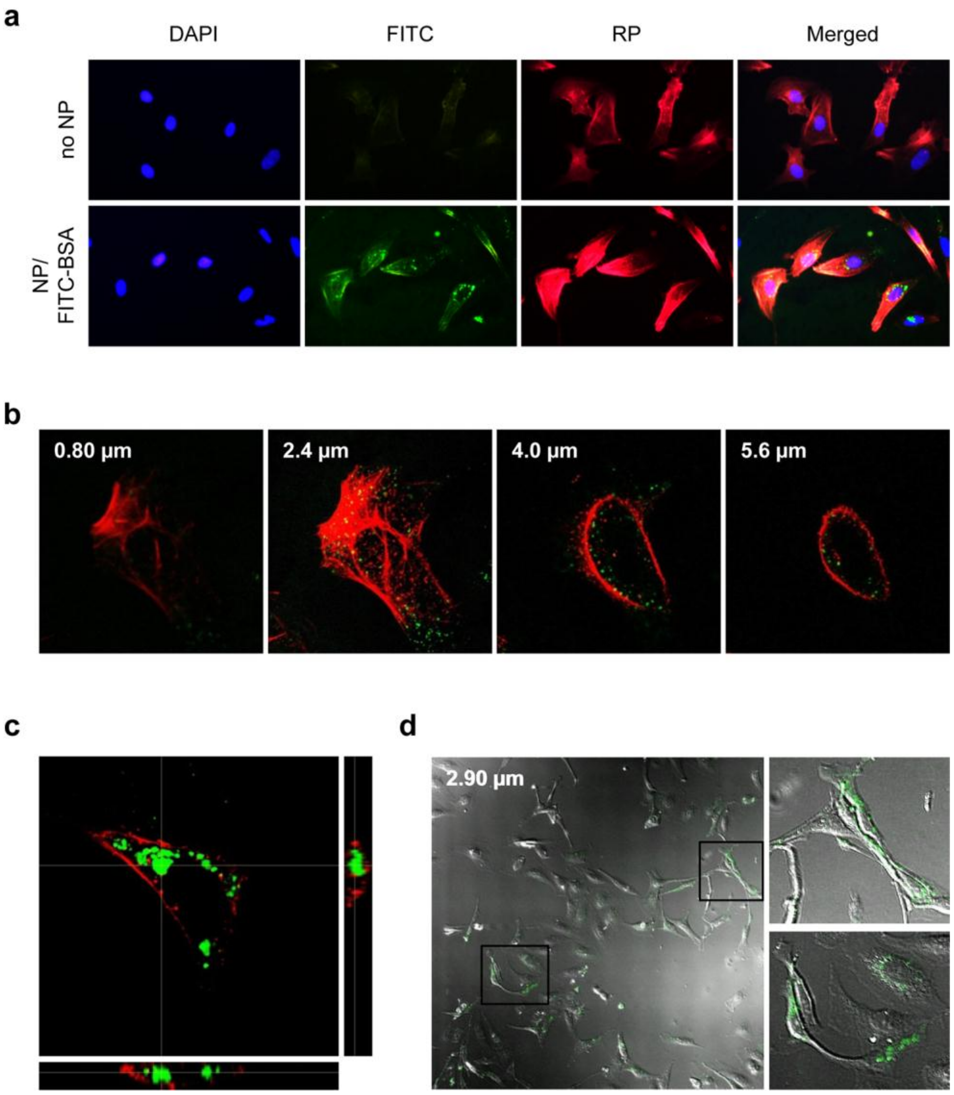

Supplementary Figure 10. Cellular uptake of NPs by LX2. (**a**) Fluorescent images for FTIC-BSA (green), Rhodamine Phalloidin (RP, red) and DAPI (blue) in LX2 cultured with (NP/FITC-BSA) or without NPs (no NP). RP and DAPI were used in staining for actin and nuclear, respectively. (**b**) Images of confocal microscopy for showing cellular uptake of NPs containing FITC-BSA by LX2 (488 nm excitation for NP, green color, and 548 nm excitation for RP, red color at ×100). Images were sliced at 0.8 µm increments. (**c**) Orthogonal view of a representative confocal image of LX2 taking NPs. (**d**) Images of confocal microscopy showed LX2 containing NPs (green), which were stacked to 2.90 µm sliced by 0.727 µm increments and observed at ×10. Rectangular boxes were enlarged regions in right panel.

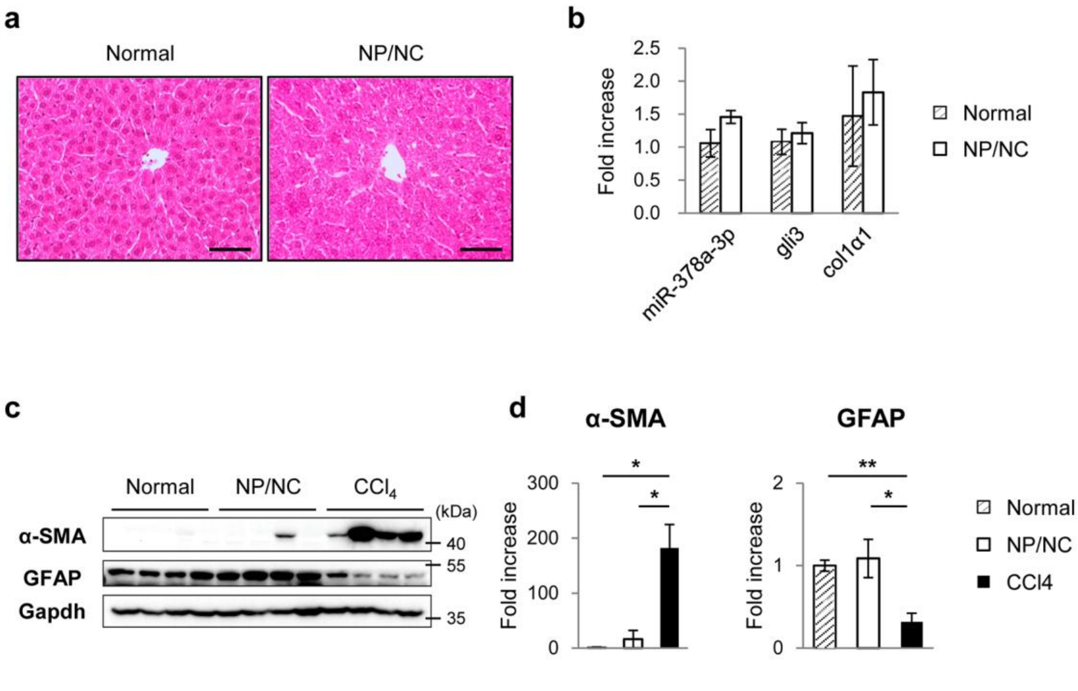

Supplementary Figure 11. Analysis of histomorphology and gene expressions between normal and NPs/NC-treated livers of mice. (**a**) Liver histomorphology was assessed by hematoxylin and eosin (H&E) staining for liver sections from representative normal and NPs/NC-treated mice (scale bar = 50 µm). (**b**) qRT-PCR analysis for *miR-378a-3p*, *gli3*, and *col1α1* expression in livers from all mice (n=4/group). Results of relative expression values are shown as mean±s.e.m.. (**c**) Western blot analysis and (**d**) cumulative densitometry analyses for α-SMA (42 kDa) and GFAP (50 kDa) in liver from all mice. GAPDH (36 kDa) was used as an internal control. Results are displayed as the mean±s.e.m. (Kruskal-Wallis test and unpaired two-sample Student's t-test, **p*<0.05, ***p*<0.005). Data shown represent one of three experiments with similar results.

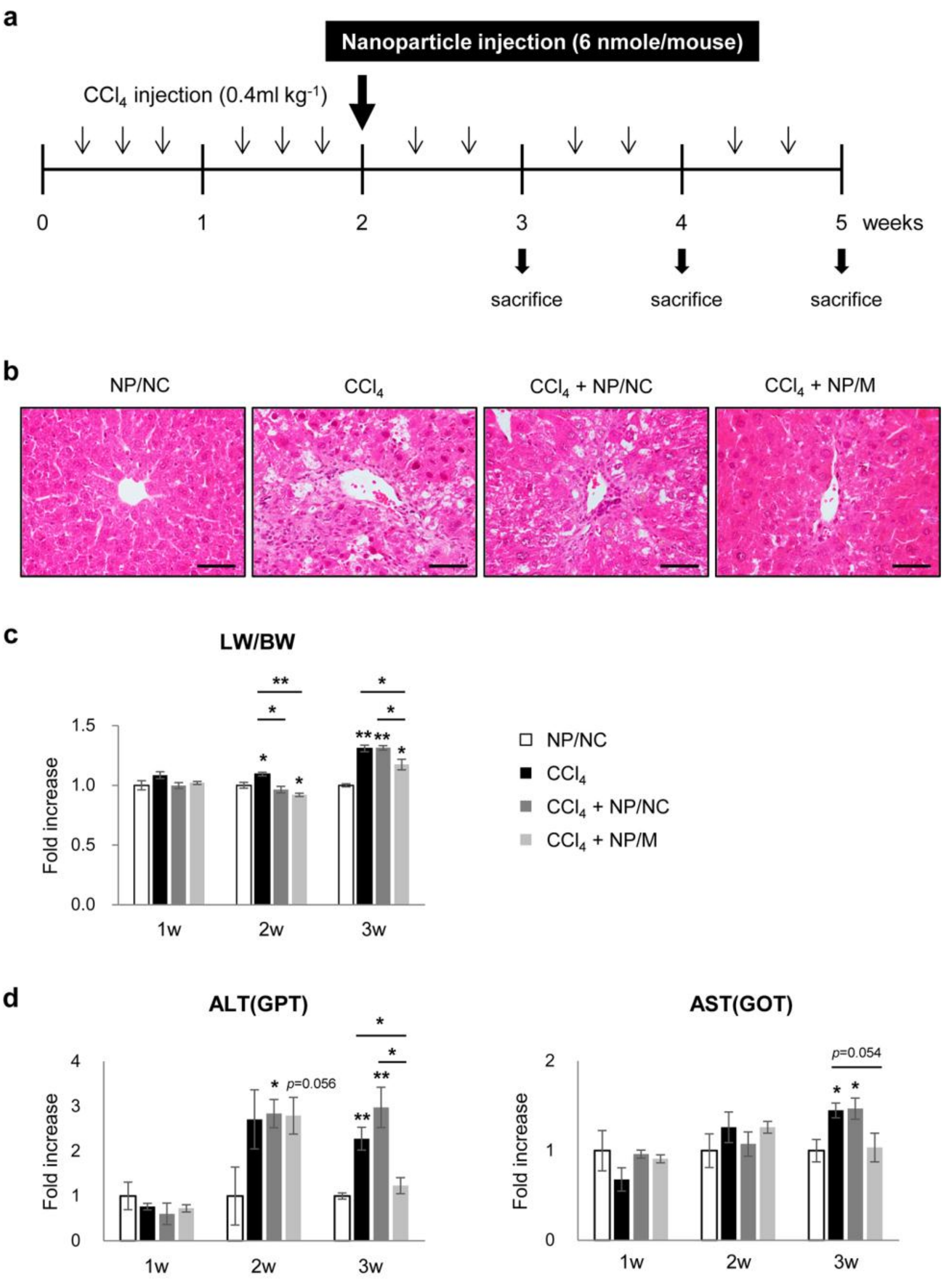

Supplementary Figure 12. MiR-378a-3p delivered by NPs influences histomorphological changes and liver function in CCl_4-treated mice. (**a**) Design of mouse experimental model. (**b**) H&E staining for liver sections from representative NP/NC, CCl_4, CCl_4+NP/NC, and CCl_4+NP/M mice at 3 weeks after NPs treatment (scale bar = 50 μm). (**c**) The ratio of LW/BW of all mice (n=4/group). Results were graphed as mean±s.e.m. (Kruskal-Wallis test and unpaired two-sample Student's t-test, **p*<0.05, ***p*<0.005 vs. NP/NC). (**d**) Relative values of serum ALT (GPT) and AST (GOT) levels of each groups were graphed as the mean±s.e.m. (Kruskal-Wallis test and unpaired two-sample Student's t-test, **p*<0.05, ***p*<0.005 vs. NP/NC).

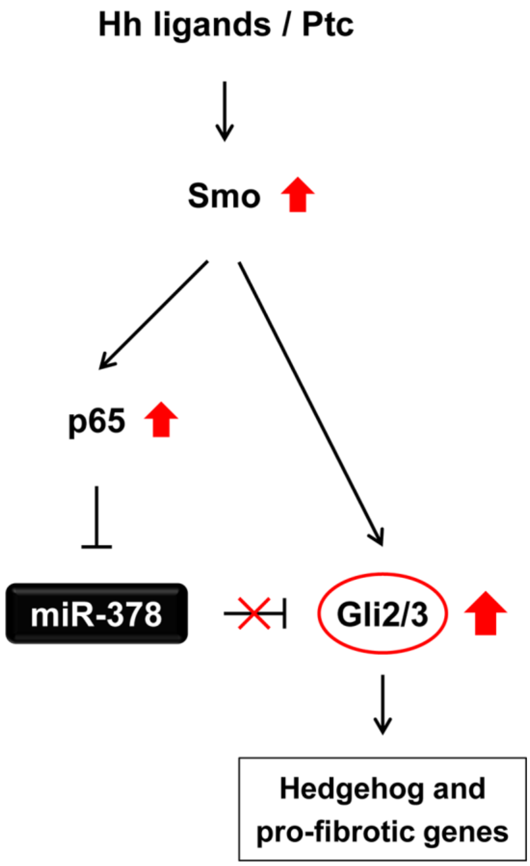

Supplementary Figure 13. A model for association of miR-378 with the Hh pathway during liver fibrogenesis. Regulation of miR-378-interacting Hh signaling in HSC was depicted. Hh ligands activate Smo, which translocates p65, Gli3, and Gli2 into nucleus. Transcription of miR-378 gene is suppressed by binding of the activated p65 with the promoter region of miR-378 gene, and decreased expression of miR-378s does not block with Gli3, eventually accelerating the expression of Hh signaling and pro-fibrotic gens in HSCs. Ptc, Patched; Smo, Smoothened; Gli, Glioblastoma.

Supplementary Figure 14. Original images of immunoblots

Figure 4b

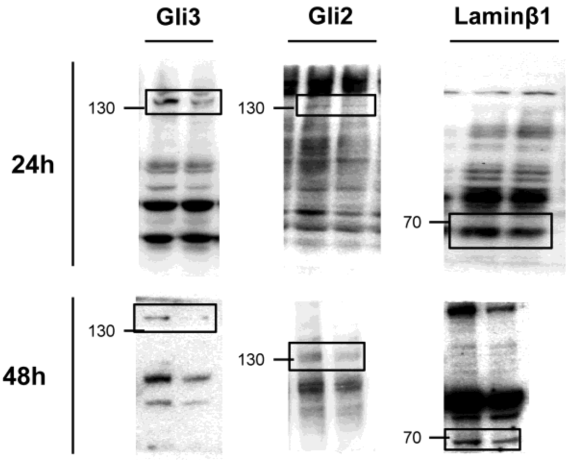

Figure 6d

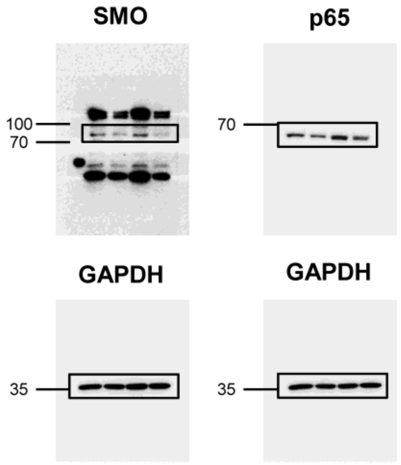

Figure 6f

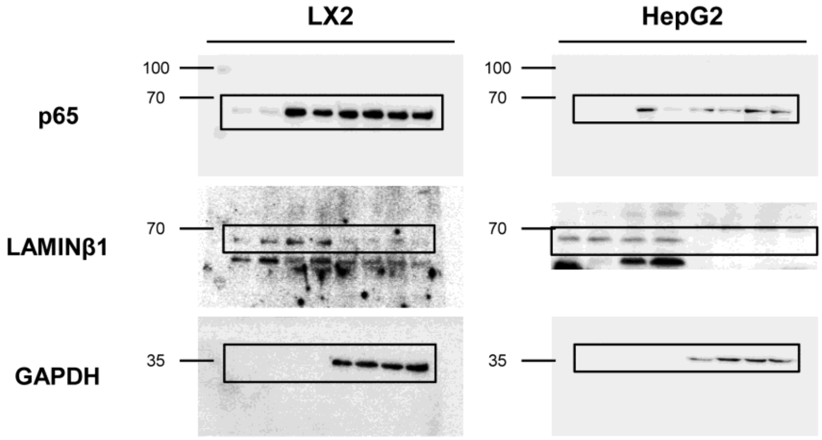

Figure 7d

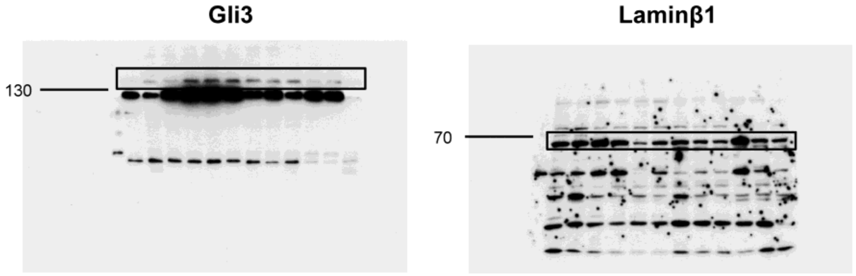

Figure 8b

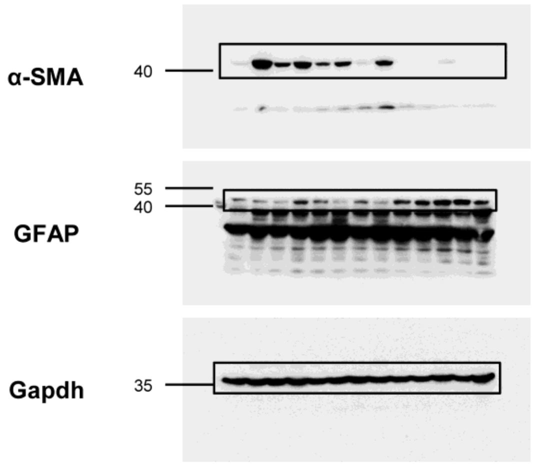

Supplementary Figure 2b

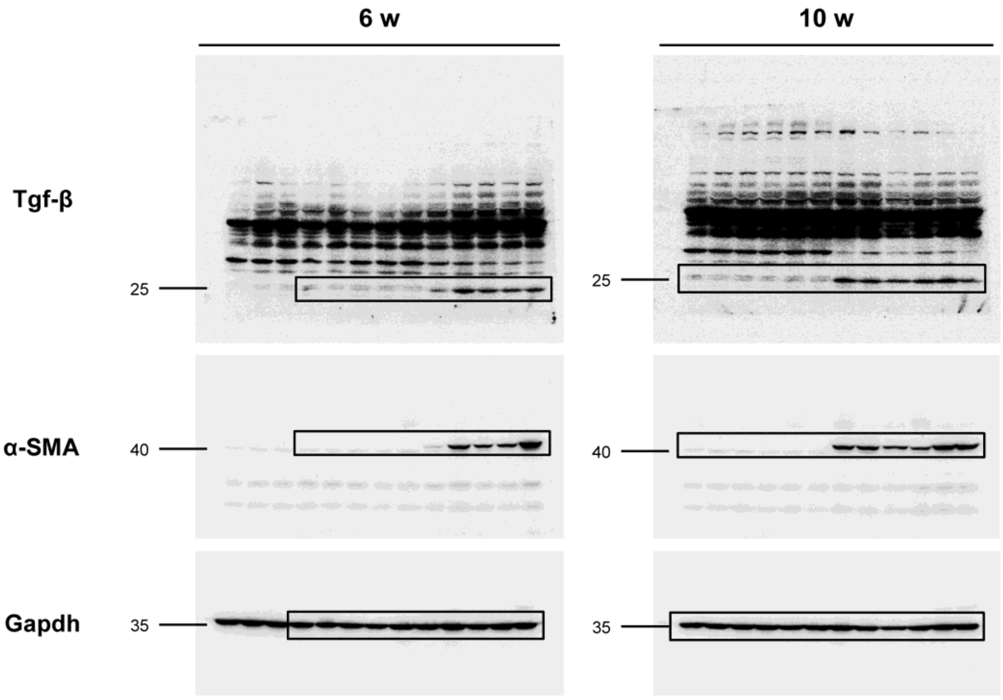

Supplementary Figure 5b

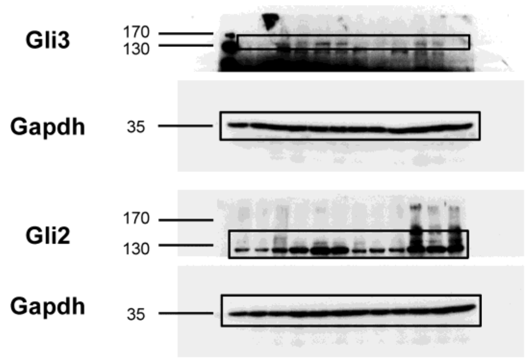

Supplementary Figure 7b

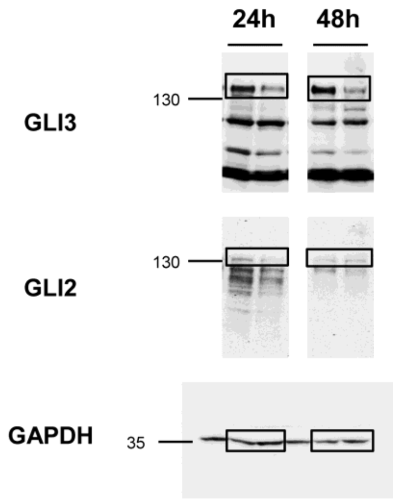

Supplementary Figure 11c

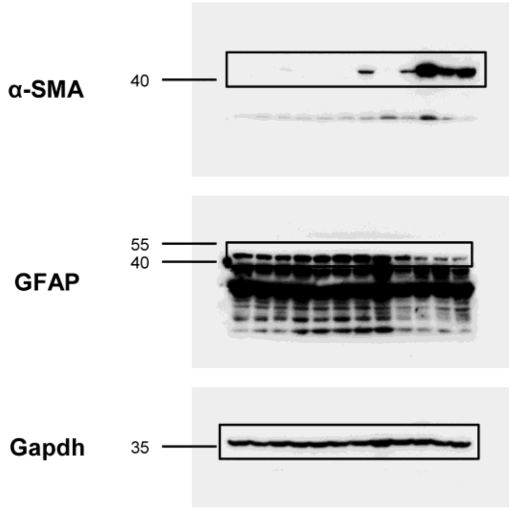

Supplementary Tables

Supplementary Table 1. Human primer sequences used for real-time qRT-PCR

Gene	Forward	Reverse
miR-378a-3p	ACTGGACTTGGAGTCAGAAGG	
miR-378b	ACTGGACTTGGAGGCAGAA	
miR-378d	ACTGGACTTGGAGTCAGAAA	
pri-miR-378a	CTCCTGACTCCAGGTCCTGT	GCCTTCTGACTCCAAGTCCA
pri-miR-378b	GGTCATTGAGTCTTCAAGG	GGTCTTTCTGCCTCCA
pri-miR-378d	ACTGTTTCTGTCCTTGTTCT	CCTGTTTCTGACTCCAAG
U1A snRNA	CGACTGCATAATTTGTGGTAGTGG	
smo	CTGGTGTGGTTTGGTTTGTG	AGAGAGGCTGGTAGGTGGTG
ptc	TCAGCAATGTCACAGCCTTC	ACTACTACCGCTGCCTGGAG
gli2	CGTGGTGCAGTACATCAAGG	CAGAGAAGCCAGTGCTTTCC
gli3	GGTGTTTGGCGCGATCAG	GAAGACACACGGGCGAGAAG
vimentin	CGAAAACACCCTGCAATCTT	GTGAGGTCAGGCTTGGAAAC
pai- I	CTCTCTCTGCCCTCACCAAC	GTGGACAGGCTCTTGGTCTG
ctgf	TCCCAAAATCTCCAAGCCTA	GTAATGGCAGGCACAGGTCT
gfap	CTGGAGGTTGAGAGGGACAA	CAGCCTCAGGTTGGTTTCAT
tgf-β	TTGACTGAGTTGCGATAATGTT	GGGAAATTGCTCGACGAT
α-sma	GTGACGAAGCACAGAGCAAA	CTTTTCCATGTCGTCCCAGT
col1α1	CAGATCACGTCATCGCACAA	TGTGAGGCCACGCATGAG
p65	CCCACGAGCTTGTAGGAAAGG	GGATTCCCAGGTTCTGGAAAC
RPS9	GACTCCGGAACAAACGTGAGGT	CTTCATCTTGCCCTCGTCCA
18S rRNA	ACACGGACAGGATTGACAGA	AGACAAATCGCTCCACCAAC

Primer sequences shown in this table were used for real-time qRT-PCR. Primers for miRNAs were used in combination with miScript Universal primer (Qiagen). All values were normalized to the level of RPS9/18S rRNA or U1A snRNA for total mRNA or miRNA, respectively.

Supplementary Table 2. Mouse primer sequences used for real-time qRT-PCR

Gene	Forward	Reverse
miR-378a-3p	ACTGGACTTGGAGTCAGAAGG	
miR-378a-5p	CTCCTGACTCCAGGTCCTGTGT	
miR-378b	CTGGACTTGGAGTCAGAAGA	
miR-378d	ACTGGCCTTGGAGTCAGAAGGT	
pri-miR-378a	AGGTCCTGTGTGTTACCTC	GGCCTTCTGACTCCAA
U1A snRNA	CGACTGCATAATTTGTGGTAGTGG	
gli1	TGTGTGAGCAAGAAGGTTGC	ATGGCTTCTCATTGGAGTGG
gli2	CAAGCAGAACAGCGAGTCAG	CCTCAGCCTCAGTCTTGACC
gli3	GCAACCTCACTCTGCAACAA	CCTTGTGCCTCCATTTTGAT
α-sma	AAACAGGAATACGACGAAG	CAGGAATGATTTGGAAAGGA
vimentin	GCTTCTCTGGCACGTCTTGA	CGCAGGGCATCGTTGTTC
col1α1	GAGCGGAGAGTACTGGATCG	GCTTCTTTTCCTTGGGGTTC
timp-1	CCTTGCAAACTGGAGAGTGACA	AAGCAAAGTGACGGCTCTGGT
tgf-β	TTGCCCTCTACAACCAACACAA	GGCTTGCGACCCACGTAGTA
mmp9	CGTCGTGATCCCCACTTACT	AACACACAGGGTTTGCCTTC
gfap	GCTTCCTGGAACAGCAAAAC	ATCTTGGAGCTTCTGCCTCA
p65	CTGATGTGCATCGGCAAG	TGCTGGGAAGGTGTAGGG
RPS9	CTTCATCTTGCCCTGGTCCA	GACTCCGGAACAAACGTGAGGT

Primer sequences shown in this table were used for real-time qRT-PCR. Primers for miRNAs were used in combination with miScript Universal primer (Qiagen). All values were normalized to the level of RPS9 or U1A snRNA for total mRNA or miRNA, respectively.
